# Loading of CAR‐T cells with magnetic nanoparticles for controlled targeting suppresses inflammatory cytokine release and switches tumor cell death mechanism

**DOI:** 10.1002/mco2.70039

**Published:** 2025-01-05

**Authors:** Felix Pfister, Lucas R. Carnell, Lisa Löffler, Philipp Boosz, Niels Schaft, Jan Dörrie, René Stein, Malte Lenz, Erdmann Spiecker, Christian M. Huber, Sami Haddadin, Carola Berking, Christoph Alexiou, Christina Janko

**Affiliations:** ^1^ Department of Otorhinolaryngology Head and Neck Surgery Section of Experimental Oncology and Nanomedicine (SEON) Else Kröner‐Fresenius‐Stiftung Professorship Universitätsklinikum Erlangen Erlangen Germany; ^2^ Friedrich‐Alexander‐Universität Erlangen‐Nürnberg (FAU) Erlangen Germany; ^3^ Department of Dermatology Universitätsklinikum Erlangen Erlangen Germany; ^4^ Comprehensive Cancer Center Erlangen European Metropolitan Area of Nuremberg (CCC ER‐EMN) Erlangen Germany; ^5^ Deutsches Zentrum Immuntherapie (DZI) Erlangen Germany; ^6^ Institute of Micro‐ and Nanostructure Research & Center for Nanoanalysis and Electron Microscopy Interdisciplinary Center for Nanostructured Films Friedrich‐Alexander‐University Erlangen Erlangen Germany; ^7^ Institute of Microwave and Photonics Friedrich‐Alexander‐Universität Erlangen‐Nürnberg (FAU) Erlangen Germany; ^8^ Chair of Robotics Science and Systems Intelligence Munich Institute of Robotics and Machine Intelligence (MIRMI), TUM School of Computation, Information and Technology Technical University of Munich Munich Germany

**Keywords:** adoptive T cell therapy, cancer, CAR‐T cell, magnetic cell targeting, pyroptosis, superparamagnetic iron oxide nanoparticles (SPIONs)

## Abstract

Therapies against hematological malignancies using chimeric antigen receptors (CAR)‐T cells have shown great potential; however, therapeutic success in solid tumors has been constrained due to limited tumor trafficking and infiltration, as well as the scarcity of cancer‐specific solid tumor antigens. Therefore, the enrichment of tumor‐antigen specific CAR‐T cells in the desired region is critical for improving therapy efficacy and reducing systemic on‐target/off‐tumor side effects. Here, we functionalized human CAR‐T cells with superparamagnetic iron oxide nanoparticles (SPIONs), making them magnetically controllable for site‐directed targeting. SPION‐loaded CAR‐T cells maintained their specific cytolytic capacity against melanoma cells expressing the CAR‐specific antigen chondroitin sulfate proteoglycan (CSPG4). Importantly, SPIONs suppressed cytokine release in the loaded CAR‐T cells, shifting the cell death phenotype in the tumor cells from pyroptosis to apoptosis. Furthermore, SPION‐loaded CAR‐T cells could be enriched in a dynamic flow model through an external magnetic field and be detected in MRI. These results demonstrate that lytic cytotoxicity is retained after SPION‐functionalization and provides a basis for future site‐specific immunotherapies against solid tumors with reduced systemic adverse side effects.

## Graphical abstract

CAR‐T cells were functionalized with superparamagnetic iron oxide nanoparticles (SPIONs) to enable site‐directed targeting of solid tumors using an external magnetic field. The SPION‐loaded CAR‐T cells maintained their killing capacity against melanoma cells expressing the CAR‐specific antigen chondroitin sulfate proteoglycan (CSPG4). Furthermore, the SPION‐loading also suppressed the cytokine release of the CAR‐T cells and switched the cell death phenotype of the tumor cells.

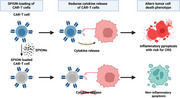

## 1 INTRODUCTION

Cancer represents an enormous burden to society, with increasing numbers patient numbers worldwide.^1^ However, recent years have seen the development of highly efficient immune therapies. Cytotoxic T cells, capable of killing malignant cells, are central in numerous strategies using the body's immune system fighting cancer, including new pillars in treatment, such as chimeric antigen receptor (CAR) T cells.^2^ In adoptive CAR‐T cells therapy, T lymphocytes are genetically engineered to express CARs that recognize and kill tumor cells.

For this purpose, autologous T cells extracted from the patient by leukopheresis are ex vivo genetically engineered, expanded, and reinfused.^3^, ^4^ CAR‐T cells have demonstrated great potential, particularly in treating hematological malignancies, where circulating target cells are easily accessible after intravenous injection. So far, six CAR‐T cell immunotherapies have been approved, all for the treatment of blood cancers, including lymphomas, some forms of leukemia, and, most recently, multiple myeloma,^5^ targeting CD19 on B cells or the B cell maturation antigen on plasma cells.^6^ However, the therapeutic success of CAR‐T cells targeting solid tumors has been limited due to various difficulties such as inefficient extravasation out of the blood vessel, ineffective trafficking of CAR‐T cells to the tumor sites, or insufficient tumor infiltration.^7^

A CAR consists of a tumor antigen‐binding moiety, typically from a single‐chain variable fragment, and one or more signal transduction domains that mediate T cell activation, proliferation, and antitumor responses. Normally, T cell activation requires antigen recognition through the T cell receptor (TCR), with the antigen presented as peptides on the major histocompatibility complex (MHC) on the target cell surface. CAR‐T cells, however, can bypass MHC restriction and include costimulatory domains, resulting in strong activation and powerful antitumor responses, but also difficult to control.^8^ Thus, CAR‐T cell therapy can lead to serious adverse side effects, such as neurotoxicity, tumor lysis syndrome, as well as on‐target/off‐tumor toxicities, where nonmalignant tissues are attacked that share the antigen.^9^ The therapy can also lead to massive, life‐threatening release of cytokines (“cytokine storm”) by CAR‐T cells and activated macrophages.^10^, ^11^ Current research focuses on minimizing toxicities while maintaining antitumor efficacy.^11^ Systemic toxicity induced by CAR‐T cells is managed through nonspecific pharmacological immune suppression or selective depletion of CAR‐T cells using “elimination” or “suicide” genes.^10^ Therapy specificity can further be improved by introducing two CAR constructs that require corecognition of the antigen for activation.^12^, ^13^ In addition, generation of CAR‐T cells through mRNA electroporation, resulting a transient CAR expression, increases safety as well.^14^

To reduce side effects and to improve the accumulation at target sites, superparamagnetic iron oxide nanoparticles (SPIONs) have come into focus. SPIONs can act as contrast agent for magnet resonance imaging (MRI), X‐ray tomography, and microcomputed tomography, as well as magnetically guidable carriers or tools to control cells through magnetic forces.^15^, ^16^, ^17^ Magnetized cells have been used for the magnetic seeding or accumulation of endothelial cells, stem cells, or dendritic cells.^18^, ^19^, ^20^ Magnetic labeling has also been applied to lymphocytes and CAR‐T cells to track their migration using MRI.^21^ SPION‐functionalized lymphocytes have demonstrate their magnetic control,^22^, ^23^, ^24^, ^25^, ^26^, ^27^, ^28^, ^29^, ^30^, ^31^ and T cells functionalized with SPIONs and PD‐1 antibody have synergistically reduced tumor growth in mice.^26^

Primary melanoma tumors and cutaneous metastases represent superficial targets, making them more accessible for magnetic T cell accumulation. Unlike hematological tumors, solid tumor‐specific antigens are scarce, increasing the risk of CAR‐T cell attacking healthy cells. The cell surface antigen chondroitin sulfate proteoglycan 4 (CSPG4) is expressed on 90% of melanoma tumors and metastases, as well as on sarcomas, astrocytomas, gliomas, neuroblastomas, and leukemia.^32^, ^33^, ^34^, ^35^ Although CSPG4 expression is 100‐fold higher on melanoma cells, it is also present on healthy tissues like chondrocytes, smooth muscle cells, cells of the neuromuscular synapse, and fetal melanocytes,^36^, ^37^, ^38^, ^39^ as well as epidermal cell precursors and hair follicles.^40^, ^41^ Therefore, CAR‐T cells targeting CSPG4 may cause severe side effects if distributed systemically. In our previous studies, a CSPG4‐specific CAR was developed, enabling antigen‐specific melanoma cell lysis and release of proinflammatory cytokines.^42^

Here, we functionalized these melanoma‐specific CAR‐T cells with SPIONs to make them magnetically controllable for tumor targeting. SPION‐loading suppressed cytokine release by the CAR‐T cells while preserving their cell‐killing capacity. Importantly, SPIONs switched the cell death phenotype in the target cells induced by the nanoparticle‐loaded CAR‐T cells from inflammatory pyroptosis to noninflammatory apoptosis, potentially reducing systemic side effects caused by excessive inflammatory immune responses during pyroptosis.

## 2 RESULTS

### 2.1 SPIONs are taken up in a dynamin‐dependent manner and localized on the membrane or intracellular vesicles

**FIGURE 1:**
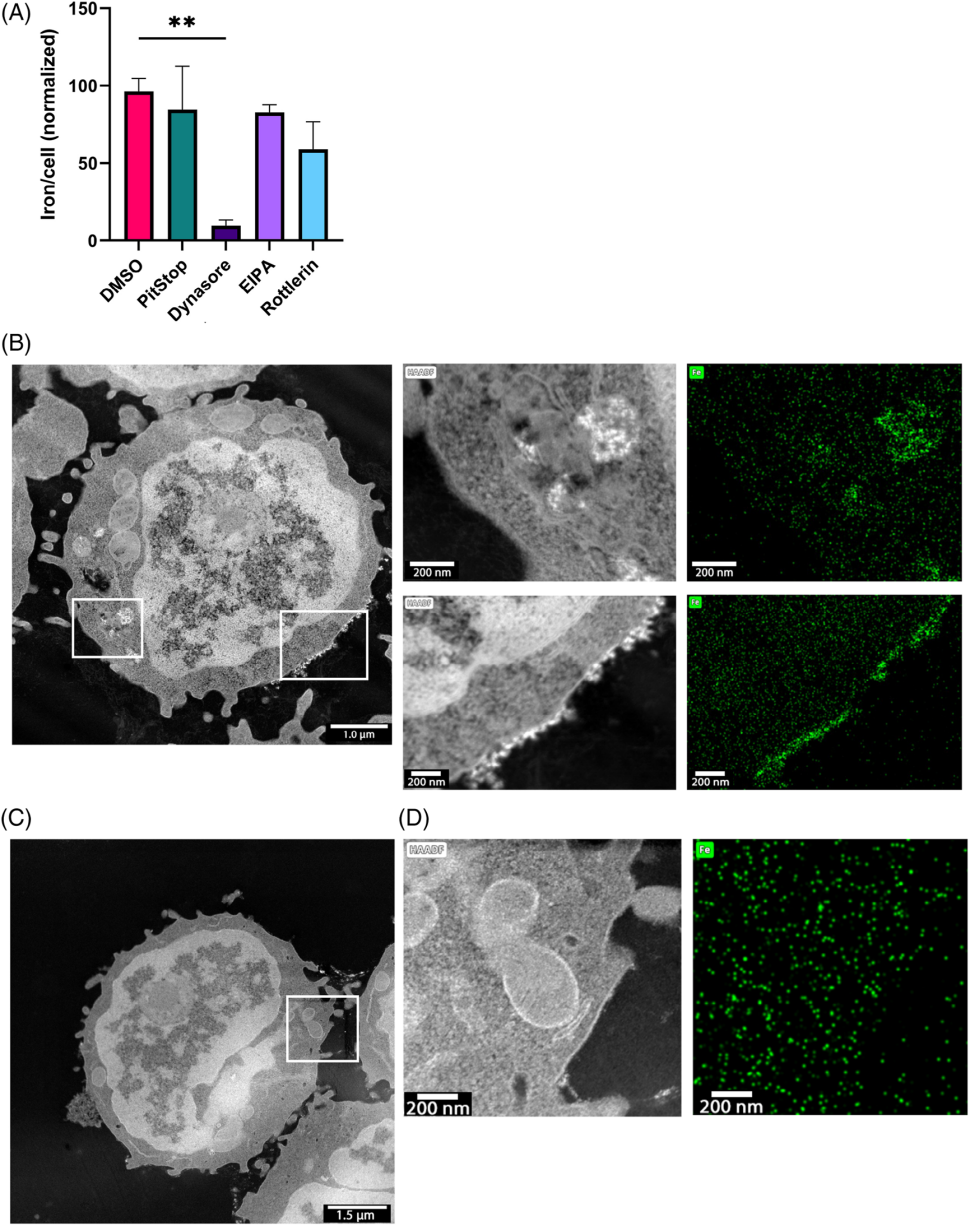
Endocytic pathway for SPION uptake and endocytic pathway and cellular localization. Primary human CD3+ T were isolated from whole blood of healthy donors loaded with 80 µg Fe/mL SPIONs. (A) The endocytic pathway of SPION uptake was investigated through the addition of the inhibitors Pitstop 2, Dynasore, EIPA, or Rottlerin. DMSO alone was used as a solvent control. Cellular iron content was analyzed by AES and normalized to the iron content of T cells which were not cultured with an Inhibitor or DMSO. (B–E) Scanning transmission electronic microscopy (STEM) images taken with high angle annular dark field (HAADF) detector giving rise to atomic number sensitive contrast (Z contrast). CD3+ T cells were loaded with 80 µg Fe/mL SPIONs overnight (C and D), dH_2_O‐treated cells served as controls (E and F). Inserts in the STEM images show the corresponding energy dispersive X‐ray spectroscopy (EDXS) mappings for iron highlighted in green in the scanned areas (right) (D and F). The white bar depicts 1 µm (D), 1.5 µm (F), or 200 nm (E and G). Cellular iron content was obtained from experiments with three individual donors. Significances (**p < 0.01; *n* = 3) were calculated using an ordinary one‐way ANOVA.

T cells can be controlled by an external magnetic field when loaded with SPIONs. We have previously shown that the amount of cellular SPIONs correlates with controllability, so maximizing loading efficiency is essential.^31^ Nanoparticles are taken up via different endocytic pathways depending on size and charge, but T cells have limited uptake, being nonactive scavengers.^43^ We analyzed SPION uptake by specifically inhibiting endocytic pathways (Figure 1A). The strongest reduction of SPION uptake was observed with dynamin (Dynasore) inhibition, whereas inhibition of macropinocytosis (EIPA and Rottlerin) and clathrin‐mediated endocytosis (Pitstop) did not affect SPION uptake. Scanning transmission electron microscopy (STEM) and energy‐dispersive X‐ray spectroscopy (EDX) revealed SPIONs on the plasma membrane and within intracellular vesicles of CD3^+^ T cells (Figure 1B,C), while no particles were detected in untreated controls (Figure 1D,E). SPIONs were identified in STEM imaging by their shape and atomic number sensitive contrast (Z‐contrast), which is characteristic for the HAADF mode (Figure 1C,E). We also observed a clear correlation between SPION position in STEM and EDXS mapping, whereas only random background was found in areas or cells without SPIONs.

To generate melanoma‐targeting CAR‐T cells, we electroporated mRNA encoding a CAR specific for CSPG4 into human CD3^+^ T cells. Mock‐electroporation without mRNA served as control. After electroporation, 80 µg Fe/mL SPIONs were added to the T cells for 4 h. In prior studies we observed that electroporation of T lymphoblasts increases cellular SPION content.^31^ Similarly, the cellular iron content of both Mock‐ and CAR‐T cells was increased in the course of generating CSPG4‐targeting CAR‐T cells through electroporation compared with nonelectroporated T cells (Figure S1A). After incubation with SPIONs, unelectroporated (UE) T cells had an iron quantity of 0.65 pg Fe/cell, whereas Mock and CAR‐T cells showed higher values of 0.95 and 1.01 pg Fe/cell, respectively. Next, we assessed the influence of SPION‐loading onbia T cell viability after electroporation by Annexin A5 (AxV) and propidium iodide (PI) staining. In contrast to our previous studies,^22^, ^25^ the loading process with SPIONs did not decrease T cell viability (Figure S1B).

In summary, SPIONs were endocytosed in a dynamin‐dependent but clathrin‐independent pathway. In TEM images, we observed that SPIONs were either bound to the membrane or were located in intracellular vesicles.

### 2.2 CAR‐T cells retain antigen‐specific tumor cell lysis after SPION‐loading

**FIGURE 2:**
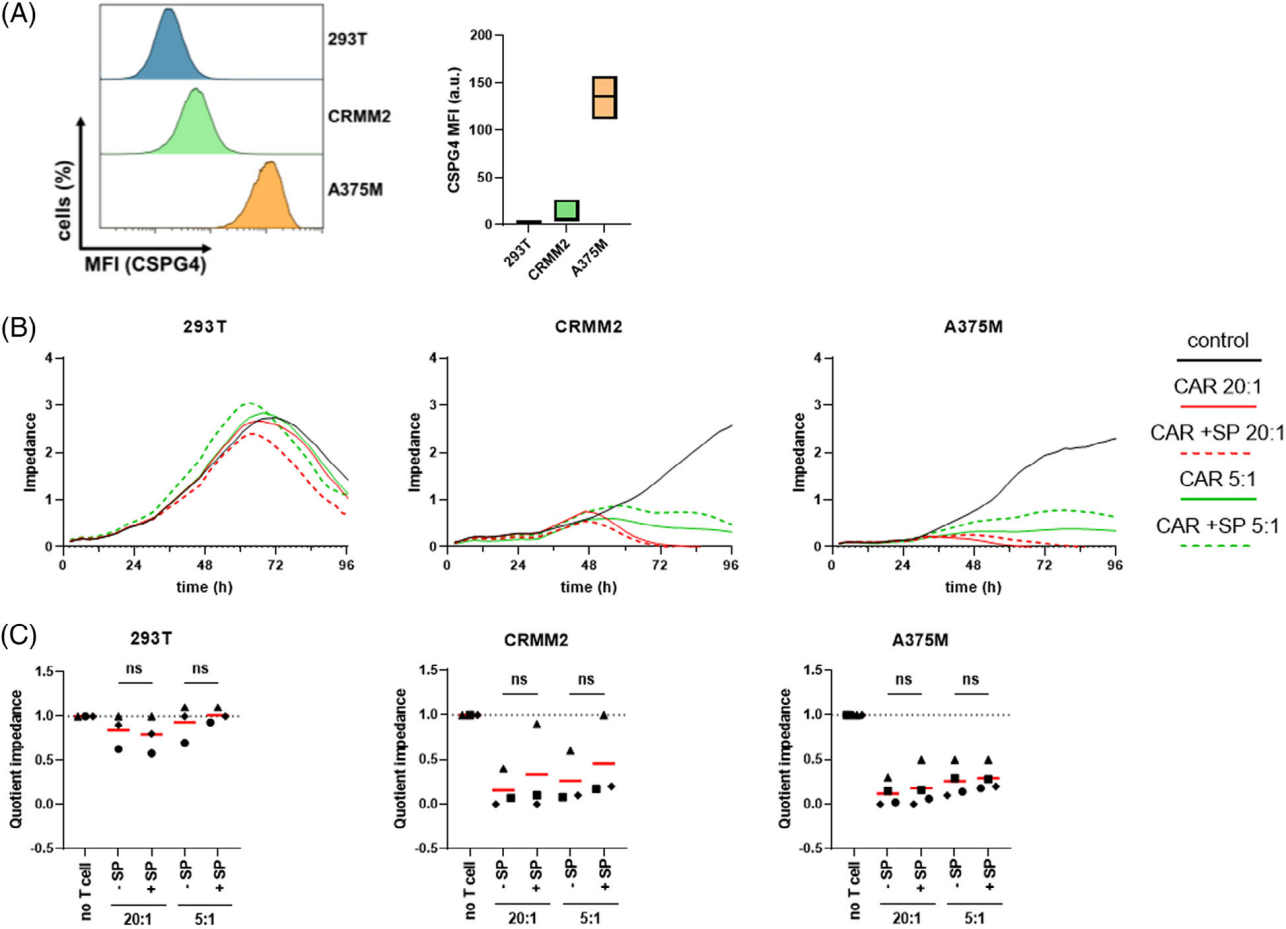
CSPG4‐specific melanoma cell lysis by SPION‐loaded CAR‐T cells. Isolated primary CD3^+^ T cells were electroporated with mRNA encoding a CSPG4‐specific CAR. Afterward, T cells were loaded with 80 µg Fe/mL SPIONs (+SP) for 4 h or dH_2_O as vehicle control (−SP). Subsequently, the T cells were coincubated with melanoma cells at ratios of 20:1 or 5:1. (A) Expression of CSPG4 on tumor cell lines, shown as mean fluorescence intensity (MFI). Surface expression was determined by staining for CSPG4 and analyzed in flow cytometry. (B) Killing of 293T, CRMM2, or A375M cells by CAR‐T cells were monitored via xCelligence real‐time impedance measurement. Cell killing kinetics of tumor cells incubated with T cells from one exemplary donor with high killing efficacy are shown. (C) To compare the killing efficacies between T cells of various donors, we used the time point, where the untreated control cells reached confluency. At this time point, a quotient was calculated between the impedance of the CAR‐T cell‐treated samples and the controls. Data were obtained in independent experiments in duplicates with T cells from three donors (for 293T and CRMM2), or with four experiments from four donors (for A375M). Every symbol represents a blood donor, the red lines are the mean values of all donors. Significances (ns, nonsignificant; (C) 293T, CRMM2: *n* = 3, A375M: *n* = 4) were calculated using a two‐way ANOVA. SP, SPIONs.

Investigating antigen‐specific targeting and subsequent tumor cell lysis is crucial for determining CAR‐T cell efficacy. However, little is known about the influence of SPIONs on CAR‐T cell effector functions. Therefore, we analyzed the ability of SPION‐loaded CAR‐T cells to bind and lyse their target cells CAR‐T cells were coincubated with cell lines varying CSPG4 expression levels, namely human embryonic kidney cells (293T) and submaxillary salivary gland carcinoma cells (A253) with no/low expression, conjunctival melanoma cells (CRMM2) and uveal melanoma cells (UPMM3) with medium expression, and amelanotic melanoma cells (A375M) expressing high quantities of CSGP4 (Figures 2A,B and S2A). Tumor cell viability was assessed using impedance‐based real‐time analysis, where increased impedance reflects increasing numbers of viable, adherent cells (cell index). Mock T cells or CAR‐T cells with or without SPION‐loading were cocultured with tumor cells at a 20:1 or 5:1 ratio. Tumor cell killing efficacy was quantified by the cell index of untreated tumor cells after 96 h (Figures 2C and S2A). Tumor cells without CAR‐T cells grew until confluency before detaching due to space and nutrient limitations. 293T and A253 cells were not affected by the coincubation with CAR‐T cells, while the viability of CRMM2 and UPMM3, and of A375M decreased proportionally to the CAR‐T cell number. A slight, but not significant decrease in the mean killing efficacy was observed after SPION‐loading, especially in tumor cells with medium CSPG4 expression. While SPIONs had no influence on tumor lysis efficacy of T cells from some donors, they diminished tumor cell killing in others.

### 2.3 SPION‐loading restricts antitumor efficacy in lower mRNA concentration

**FIGURE 3:**
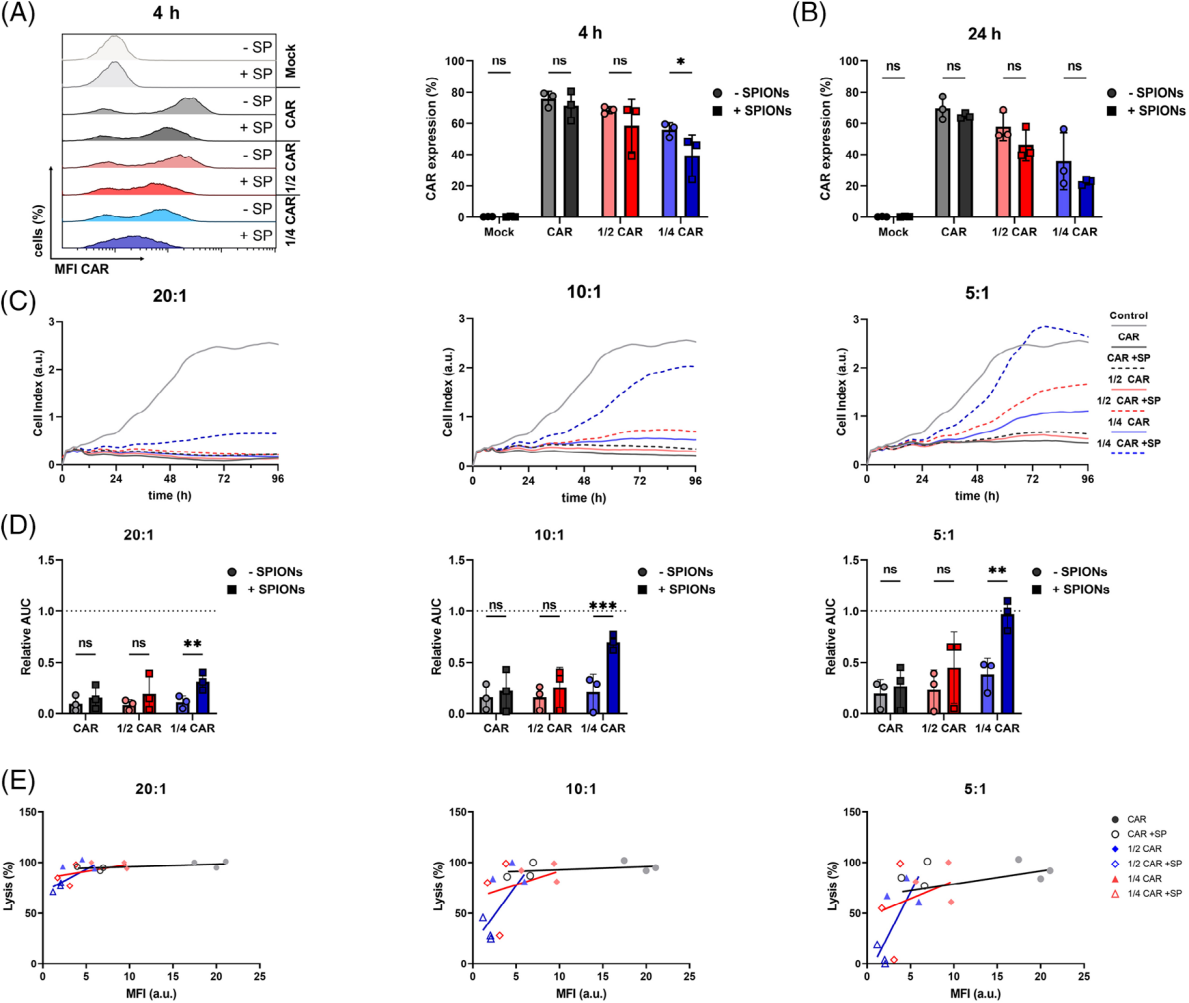
Tumor cell lysis depends on CAR expression density on T cells. Isolated CD3^+^ T cells were electroporated without mRNA (Mock) or mRNA encoding a CSPG4‐specific CAR (CAR), either with 150 µg/mL, 75 µg/mL (1/2), or 37.5 µg/mL (1/4). T cells were incubated with 80 µg Fe/mL SPIONs (+SPIONs) for 4 h, or dH_2_O as vehicle control (−SPIONs). (A and B) Expression of CAR on T cells, which had been electroporated with various amounts of mRNA and were loaded or nonloaded with SPIONs, after 4 h (A) or 24 h (B) as determined by antibody staining in flow cytometry. (C–E) A375M cells were treated with Mock or CAR‐T cells at different tumor to T cell ratios (1:5, 1:10, 1:20). Killing of A375M cells was monitored via xCelligence real‐time impedance measurement. Cell killing kinetics of tumor cells incubated with T cells from one exemplary donor are shown. (D) To compare the killing efficacies between T cells of various donors, we used the time point, where the untreated control cells reached confluency. At this time point, a quotient was calculated between the Area under the curve (AUC) of the CAR‐T cell‐treated samples and the controls. (E) Correlation of tumor cell lysis and CAR expression on T cells. Experiments were performed with T cells isolated from three donors. Significances (ns, nonsignificant; *p ≤ 0.05; **p < 0.01; ***p < 0.001; *n* = 3) were calculated using a two‐way ANOVA.

CAR‐T cells efficacy is influenced by both antigen density on the tumor cells and CAR density on the T cell.^44^ High CAR density on the T cell is crucial for antigen recognition and successful tumor lysis.^45^ To analyze the influence of SPION‐loading on CAR expression robustness, CAR mRNA concentration were reduced to ½ and ¼ of the established 150 µg/mL.^42^, ^46^ After 4 h and 24 h of SPION‐loading, residual CAR expression on CAR‐T cells was analyzed. SPIONs did not significantly affect the percentage of CAR‐expressing T cells at maximum mRNA dose, but they reduced CAR‐positive cell percentages under reduced mRNA conditions compared with controls (Figure 3A,B). Additionally, SPIONs lowered the CAR expression density (reflected by MFI) in all cases.

The killing capacity of the CAR‐T cells against A375M cells was evaluated at ratios of 20:1, 10:1, and 5:1 and compared with nonloaded CAR‐T cells. No significant differences were observed when treating tumor cells with high CAR‐T cell numbers (20:1 or 10:1 ratios) using standard or ½ mRNA dose (Figures 3C,D and S3). However, CAR‐T cells transfected with ¼ of CAR mRNA showed a significant reduction of A375M cell lysis at all ratios, with the effect being more pronounced at 10:1 or 5:1 ratios. Plotting CAR expression against tumor cell lysis showed reduced lysis efficacy of CAR‐T cells with low CAR‐specific MFI (Figure 3E).

In summary, SPION‐loading reduced the CAR density under low mRNA conditions, leading to a fewer CAR‐positive cells and decreased tumor‐killing capacity at lower CAR‐T cell ratios.

### 2.4 Inflammatory cytokine secretion by CAR‐T cells is inhibited after SPION‐loading

**FIGURE 4:**
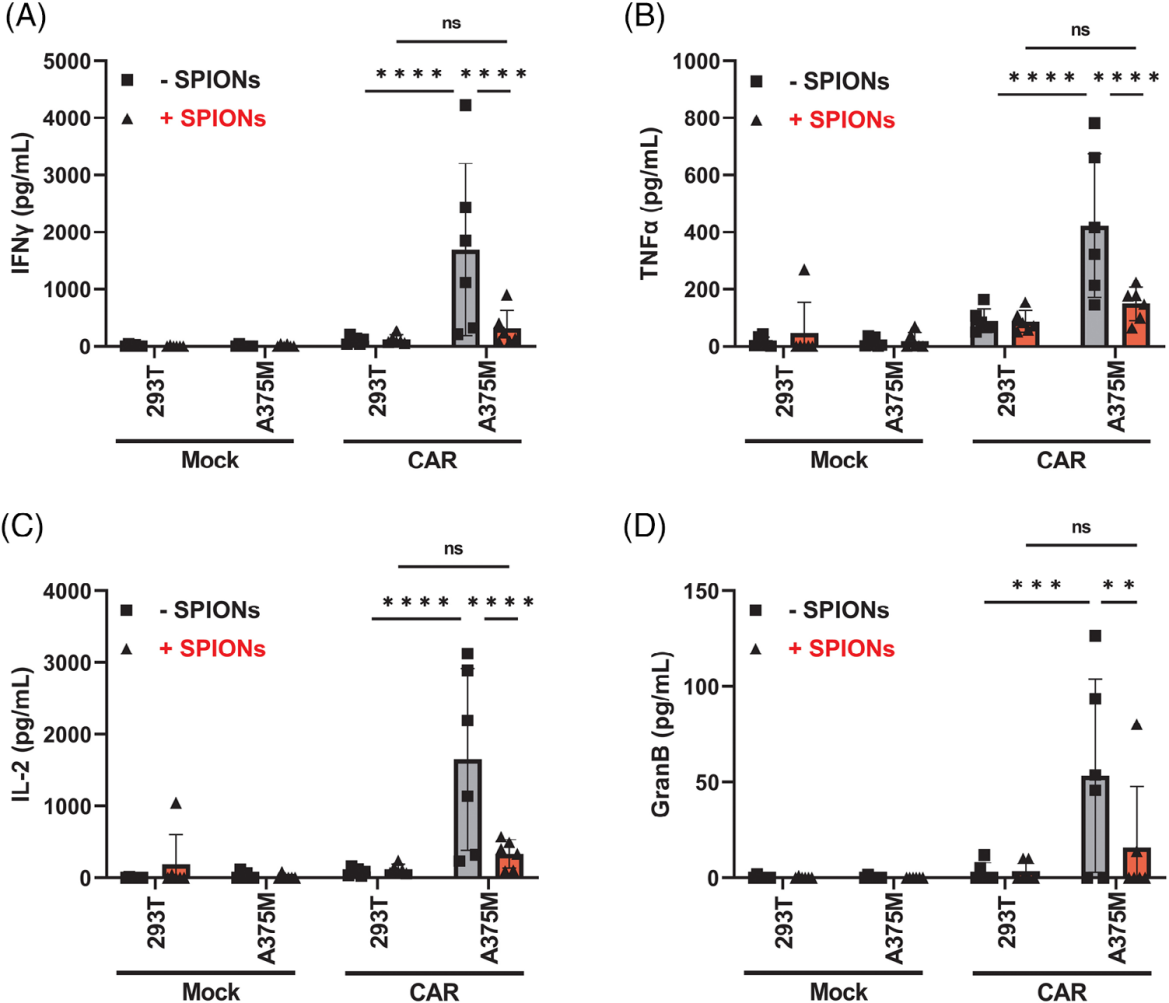
SPION‐loading results in reduced cytokine secretion by CAR‐T cells. Isolated primary CD3^+^ T cells were either electroporated without mRNA (Mock) or received mRNA encoding a CSPG4‐specific CAR. Afterward, T cells were loaded with 80 µg Fe/mL SPIONS (+SPIONs) for 4 h or dH_2_O as vehicle control (−SPIONs). Subsequently, the T cells were coincubated with the tumor cells at a ratio of 1:1 for 24 h. Then, the supernatant was harvested and analyzed for cytokine content via bead‐based multiplex assay. Experiments were performed with T cells isolated from three independent donors. Significances (ns, nonsignificant; **p < 0.01; ***p < 0.001; ****p < 0.0001; *n* = 6) were calculated using a two‐way ANOVA.

CAR‐T cell therapy is often constrained by the risk of cytokine release syndrome (CRS), a severe and potentially life‐threatening inflammatory response caused by excessive and uncontrolled cytokine secretion from activated CAR‐T cells, leading to systemic inflammation.^47^ CSPG4‐specific CAR‐T cells have previously been shown to secrete inflammatory cytokines after contact with A375M cells.^48^ In nonloaded CAR‐T cells, we observed the release IFNγ, TNFα, IL‐2, and Granzyme B after contact with A375M cells (Figure 4). No cytokine release occurred CAR‐T cells coincubated with 293T cells, or Mock T cells, which served as controls. Interestingly, SPION‐loaded CAR‐T cells exhibited significantly reduced secretion of these cytokines.

Antigen‐specific activation and proliferation were not significantly affected by SPION‐loading (Figure S4). Upon contact with A375M cells, SPION‐loaded CAR‐T cells formed proliferation clusters and expressed the proliferation marker Ki67 at similar levels to nonloaded CAR‐T cells (Figure S4A,B). Similarly, the activation marker CD25 and the effector molecule Granzyme B were upregulated to the same degree (Figure S4C,D). CAR‐T cells generally showed slight marker upregulation, potentially due to intrinsic activity of the signaling modules within the CAR (i.e., CD28, CD3ζ).^49^ Additionally, SPION‐loading did not impact the differentiation from naive to central memory, effector, and effector memory T cells, identified by the expression of CD45RO and CD197, after contact with A375M cells (Figure S4E).^50^

In conclusion, SPION‐loaded CAR‐T cells exhibited a significant reduction in cytokine release, without affecting antigen‐specific activation, proliferation, differentiation, or the intracellular cytokine content.

### 2.5 SPION‐loading switches CAR‐T cell induced tumor cell‐death

**FIGURE 5:**
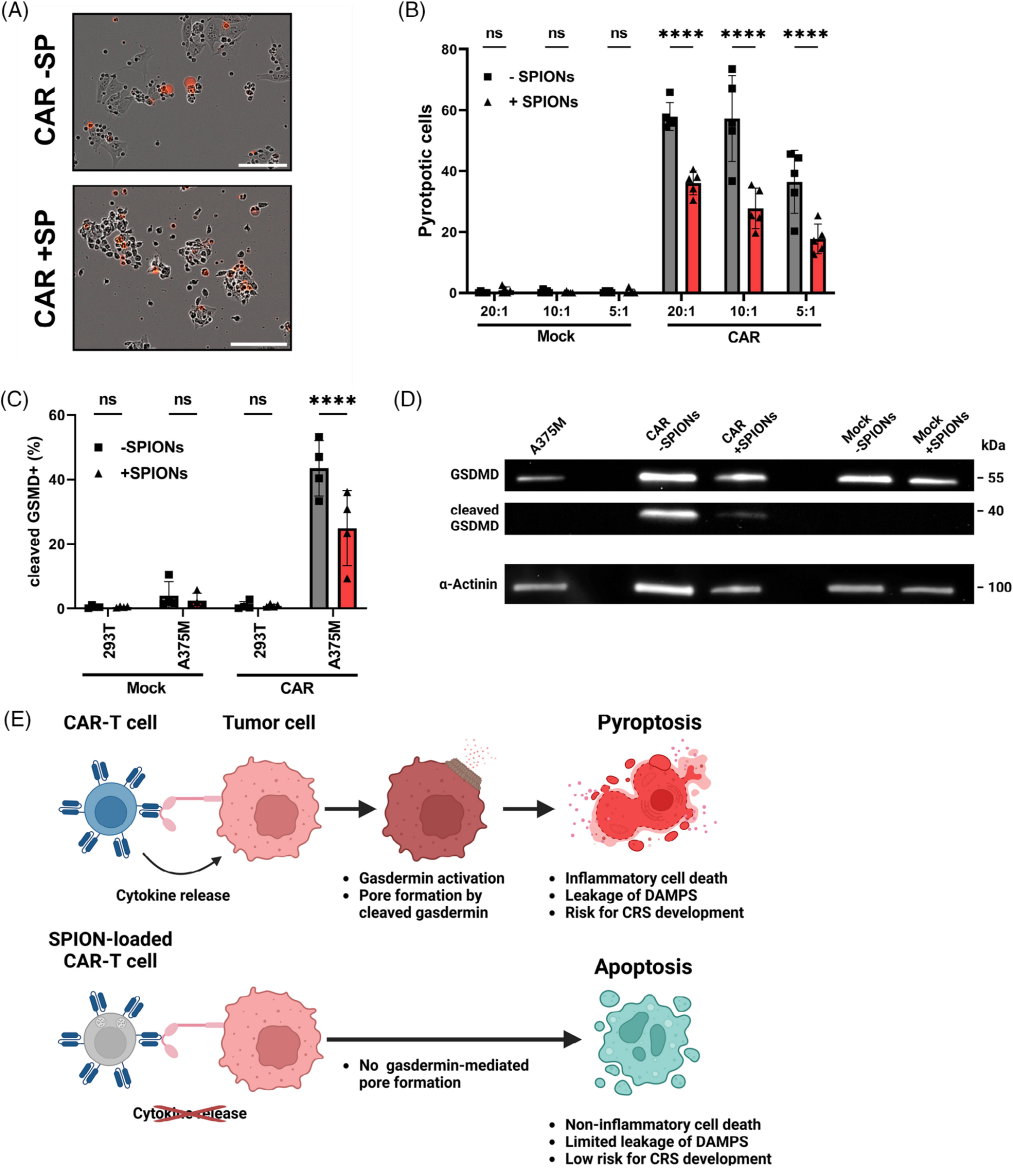
Tumor cell death phenotype is altered by SPION‐loading of CAR‐T cells. Isolated CD3+ T cells were electroporated without mRNA (Mock) or with mRNA encoding the CSPG4‐specific CAR (CAR). T cells were loaded with 80 µg Fe/mL SPIONs (+SPIONs) for 4 h, cells coincubated with dH_2_O served as vehicle control (−SPIONs). Afterward, the cells were incubated with A375M cells. (A and B) Cells were monitored in IncuCyte system in transmission and fluorescence mode. Swelling cells with one large final PI‐positive bubble are referred to pyroptotic cells, shrinking cells with multiple PI‐negative small blebs are referred to as apoptotic ones. (A) Examplatory live‐cell fluorescence microscopy pictures at 10 h after incubation. The white bar indicates 100 µm. (B) Analysis of pyroptosis frequency per well after 10 h. (C and D) Gasdermin D cleavage was investigated 4 h after coincubation. (C) The frequencies of cleaved Gasdermin D in A375M cells was determined by flow cytometry after staining for cleaved Gasdermin D, CD4, and CD8. (D) Western blotting was performed to determine cleaved Gasdermin levels after coincubation. (E) Proposed mechanism for alteration of cell death phenotype by SPION‐loading of CAR‐T cells. CAR‐T cells are loaded with SPIONs. Significances (ns, nonsignificant; *p ≤ 0.05; **p < 0.01; ****p < 0.0001; (B) *n* = 5, (C) *n* = 4) were calculated using a two‐way ANOVA. −SP, without SPIONs; +SP, with SPIONs; GSDMD, Gasdermin D.

Tumor cell pyroptosis significantly contributes to CAR‐T cell therapy related toxicities by releasing massive amounts of inflammatory DAMPs, which may lead to CRS.^51^ Pyroptosis is mediated by CAR‐T cells via the activation of caspases 1 and 3, which cleave Gasdermin E or Gasdermin D, respectively, resulting in the formation of membrane pores.^52^ Microscopic analysis revealed notable difference in tumor cell death morphology after contact with nonloaded versus SPION‐loaded CAR‐T cells (Figure 5A). Nonloaded CAR‐T cells frequently induced a large, PI positive, final bubble, characteristic of pyroptosis. In contrast, SPION‐loaded CAR‐T cells caused tumor cell shrinkage, membrane blebbing, and decomposition into smaller bodies after coincubation, indicative of apoptotic death.

Intracellular staining showed reduced caspase 3‐mediated cleavage of Gasdermin D in A375M cells coincubated with SPION‐loaded CAR‐T cells compared with nonloaded (Figure 5C). Western blot analysis confirmed lower levels of cleaved Gasdermin D in A375M cells coincubated with SPION‐loaded CAR‐T cells compared with nonloaded or Mock‐electroporated T cells (Figure 5D).

Live cell imaging using correlative epifluorescence microscopy supported these findings, showing apoptotic morphology in A375M cells treated with SPION‐loaded CAR‐T cells. After 9 h, CAR‐T cells initiated contact with A375M tumor cell (“kiss of death”), leading to membrane blebbing (11 h) and cell detachment (14 h), characteristic of apoptosis rather than pyroptosis. Mock‐electroporated T cells did not induce cell death in the observed time (Figure S7 and Video S1 and S2).

In conclusion, SPION‐loaded CAR‐T cells induced significantly less inflammatory pyroptosis in target tumor cells compared with nonloaded CAR‐T cells, promoting apoptotic cell death instead (Figure 5E).

### 2.6 Magnetic accumulation of T cells after SPION‐loading

**FIGURE 6:**
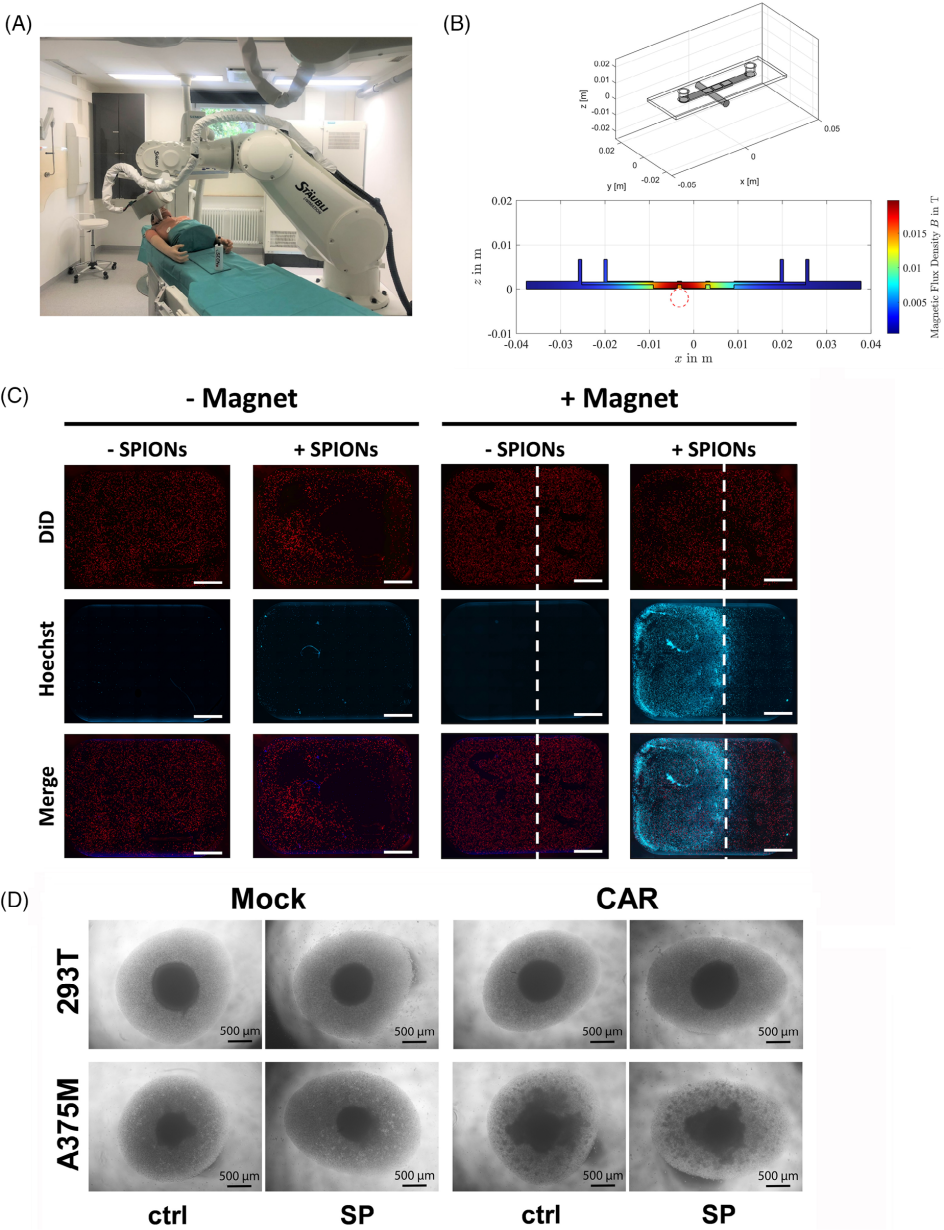
Magnetic guidance of SPION‐loaded CAR‐T cells for targeted cell death induction. (A) Magnet robot for in vivo application. (B) 3D geometry of the channel slide to magnet‐configurations. Simulation of the magnetic field for the accumulation of SPION‐loaded CAR‐T cells in channel slide. (C and D) Isolated CD3+ T cells were electroporated without mRNA (Mock) or with mRNA encoding the CSPG4‐specific CAR (CAR). T cells were loaded with 80 µg Fe/mL SPIONs (+SPIONs) for 4 h, cells coincubated with dH_2_O served as vehicle control (−SPIONs). (C) A375M cells were seeded in a monolayer in the wells of a μ‐Slide I Luer 3D and embedded in Fibrin. A bar magnet was placed underneath the µ‐slide and CA‐RT cells were then accumulated for 1 h in flow. The dottet white line indicates the borders of the magnet while the white bar indicates 1 mm. (D) A375M spheroids were coincubated with CAR‐T cells for 36 h and then analyzed by microscopy. The black bar indicates 500 µm.

Enrichment of CAR‐T cells in the tumor region is crucial for effective therapy, ensuring successful trafficking, sustained target engagement, and minimizing on‐target/off‐tumor toxicities. SPIONs enable the magnetic navigation of cells, which can be performed through an external electromagnet controlled by a robot (Figure 6A).

The ability of SPION‐loaded CAR‐T cells (labeled in blue) to being accumulated under dynamic flow conditions, simulating blood circulation, was analyzed. A375M tumor cells (labeled in red) were seeded in an extracellular matrix in the wells of an ibidi μ‐slides I Luer 3D channel slide. A neodymium bar magnet was placed under the middle well to introduce a magnetic field, simulated in Figure 6B. Magnetic accumulation of CAR‐T cells in flow was performed by pumping with 9.6 mL/min for 1 h. After flushing the slides and removal of the magnet, fluorescence microscopy confirmed that SPION‐loaded CAR‐T cells accumulated at the position of the magnet, while no accumulation could be detected of nonloaded CAR‐T cells or in wells where no magnet was added (Figure 6C). The infiltration ability of SPION‐loaded CAR‐T was assessed using of A375M and 293T spheroids. After 36 h of coincubation of CAR‐T cells with tumor spheroids at a 60:1 ratio, the compact structure of the A375M spheroids was lost and T cells formed proliferation spots within the loosened structure. Mock‐electroporated T cells neither dissolved the A375M spheroid, nor formed proliferation spots. The same occurred with CAR‐T cells incubated with 293T spheroids (Figure 6D).

In sum, SPION‐loading enabled CAR‐T cell accumulation under dynamic flow through an external magnetic field. Furthermore, SPION‐loaded CAR‐T cells retained their ability to infiltrate tumor spheroids in an antigen‐specific way.

### 2.7 MRI detection of SPION‐loaded T cells

Iron oxide nanoparticles‐based contrast agents are widely employed in T_2_‐weighted MRI applications.^53^ While SPIONs can be used to label immune cells and monitor trafficking and persistence using MRI, studies involving their use with T cells remain limited.^54^

**FIGURE 7:**
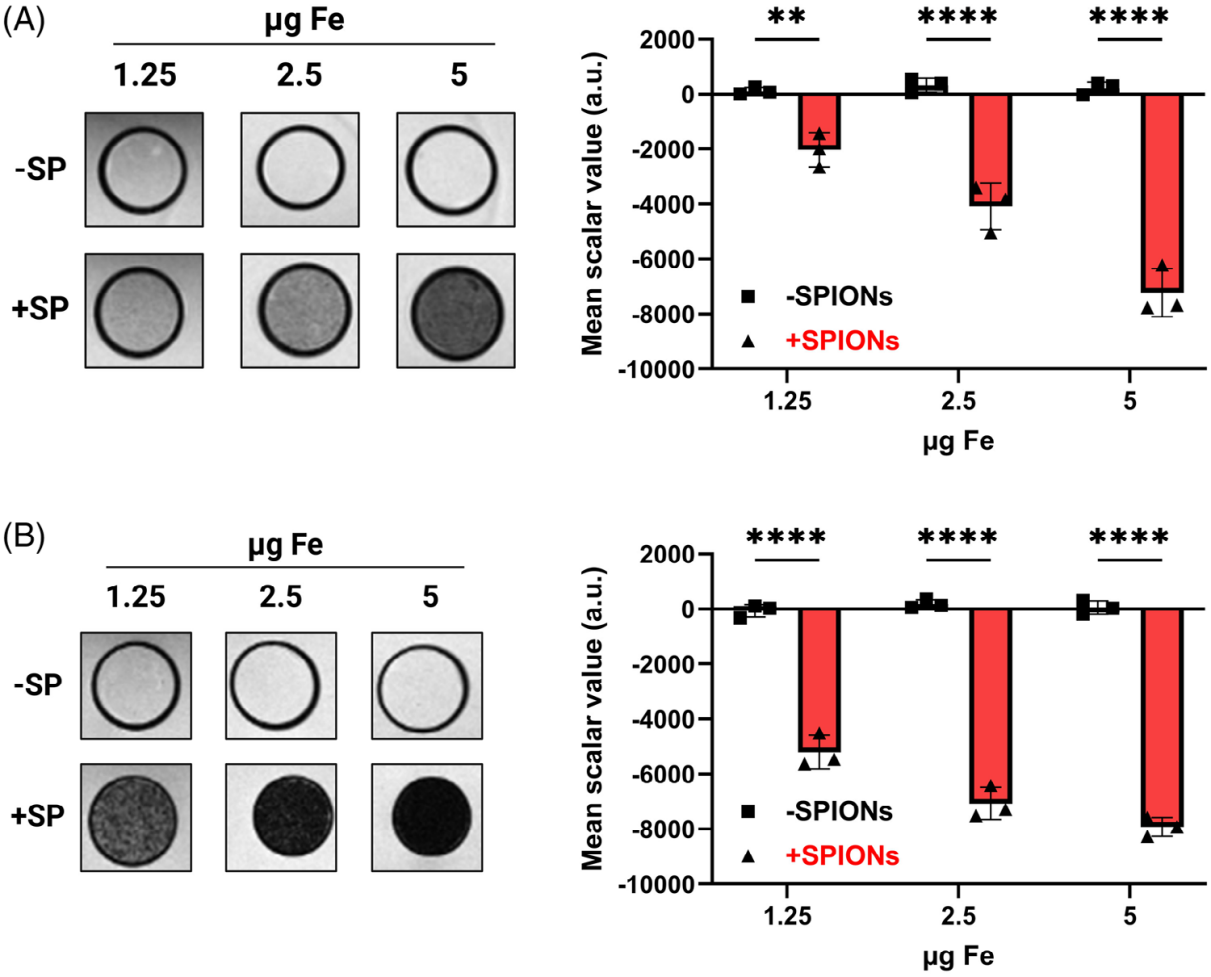
Detection of SPION‐loaded T cells though MRI. Jurkat T cells were loaded with 80 µg Fe/mL SPIONs for 16 h (+SPIONs), with controls receiving dH_2_O (−SPIONs). Then, cellular iron concentrations were detected using AES. Afterward, cells were seeded at total iron concentrations of 1.25, 2.5, and 5 µg iron in a PCR tube to which the same volume of 2% agarose was added. After solidifying, PCR tubes were placed in a 50 mL Falcon tube and embedded in 1% agarose. Afterward, the cells were analyzed by a 7 T MRI with (A) T2‐weighted and (B) 3D‐FLASH sequences. The scalar values of inside and outside of the PCR tubes were measured using the software 3D slicer. Significances (**p < 0.01; ***p < 0.001; ****p < 0.0001; *n* = 3) were calculated using a two‐way ANOVA. −SP, without SPIONs; +SP, with SPIONs.

To investigate the suitability of our SPIONs for detection of labeled cells using MRI, SPION‐loaded Jurkat T cells were suspended in agarose at iron concentrations of 5, 2.5, and 1.25 µg Fe and imaged using a 7 T MRI with T_2_‐weighted and 3D‐Fast Low Angle Shot (FLASH) imaging (Figure 7). SPION‐loading resulted in a concentration‐dependent MRI signal at all tested concentrations, while no signal was detected in nonloaded controls.

In conclusion, SPION‐loaded T cells can be detected via MRI, demonstrating the potential for in vivo detection and tracking of labeled T cells.

## 3 DISCUSSION

CAR‐T cell therapy for solid tumors remains challenging due to an insufficient tumor infiltration, hostile tumor microenvironment, and severe off‐target toxicity. To address these issues, we functionalized CAR‐T cells with citrate‐coated SPIONs to enable magnetic guidance for accumulation in the tumor region. This study demonstrated that SPION‐loading suppressed inflammatory cytokine release while preserving tumor‐lysis capability. Notably, cytokine reduction was associated by a switch in the tumor cells death from inflammatory pyroptosis to noninflammatory apoptosis, potentially mitigating systemic inflammatory side effects of conventional CAR‐T cell therapy like CRS.

The presence of tumor infiltrating T cells has been shown to improve patient prognosis and therapeutic strategies in various cancers.^55^ We have demonstrated that T cells can be magnetically guided after SPION‐loading allowing for enhanced infiltration.^22^, ^25^ However, T cell nanoparticle loading is challenging due to their small size, nonphagocytic nature, low endocytic activity, and high nucleus to cytoplasm ratio.^56^ Additionally, the limited cytosolic space in T cells restricts nanoparticle internalization, unlike in larger antigen‐presenting cells.^57^ Nanoparticle uptake primarily occurs through receptor‐mediated endocytosis, clathrin‐independent pathways, and macropinocytosis in activated T cells. For receptor‐mediated endocytosis by clathrin‐coated vesicles, nanoparticles with a diameter of around 50 nm are optimal,^58^ which aligns with the particle size used in this study. Using endocytosis inhibitors, we identified the uptake of SPIONs as dynamin‐dependent process (Figure 1A).

Previously, we demonstrated a direct correlation between the T cell magnetic guidability and cellular SPION content.^31^ Improved lymphocyte labeling with iron oxide nanoparticles was achieved via lipofectamine and electroporation, likely facilitating nanoparticle uptake by the formation of pores in the plasma membrane.^59^, ^60^, ^61^ Although SPION‐loading negatively affected T cell viability in earlier studies,^22^, ^25^ the addition of IL‐7, an essential factor for lymphoid survival, mitigated this effect (Figure S1B).^62^

Despite the negative zeta potential of the SPIONs, tight attachment of the particles to the negatively charged plasma membrane was observed, as well as intracellular uptake and no spilling onto nonloaded cells (Figure 1B,C).^22^ This contrasts other studies, in which positively charged 3‐aminopropyl‐triethoxysilane‐coated SPIONs strongly adhered to the T cell surface, yet also spilled over to nonloaded cells.^28^

In our previous studies, SPION‐loading of T cells expressing exogenous tumor‐specific TCR did not reduce cytokine release.^31^ Unlike CAR‐T cells, which recognize antigens independent of the MHC, TCR‐mediated recognition is MHC‐restricted and different in the activation pattern and sensitivity.^63^ A single TCR can react to one antigen‐presenting MHC, whereas CARs require a higher density of target molecules, although with a higher affinity.^63^ Concerning the cytokine release and cytolysis thresholds, the antigen density for the CAR must be significantly higher for cytokine release than for cytolysis.^64^ For instance, an anti‐CD20 CAR‐T cells with a CD28 intracellular domain required around 200 molecules per target cell for killing, but a few thousand for cytokine secretion.^65^ In the presence of SPIONs, the number of CARs on the T cell surface was reduced (Figure 3A,B), possibly due to endocytic vesicle formation, resulting in less sensitivity toward the target, as seen with CAR loss though trogocytosis.^66^ The different activation thresholds explain the observed divergence between lysis and cytokine release toward CSPG4‐expressing tumor cells, with continued tumor cells lysis (Figure 2), while cytokine secretion was diminished (Figure 4).

Consequently, CAR‐T cell responses like cytolysis and cytokine release may be modulated by altering their sensitivity via SPION‐loading. Additionally, CD4^+^ and CD8^+^ T cells differ in signaling and function as CAR‐T cells, with CD8^+^ CAR‐T cells having more intracellular granzyme B and perforin and kill faster than CD4^+^ CAR‐T cells.^67^ In contrast, CD4^+^ CAR‐T cells have a higher capacity to release effector cytokines such as IFNγ, TNFα, and IL‐2.^68^ Thus, different activation thresholds of the CD8^+^ and the CD4^+^ T cells may contribute to the divergence between lysis and cytokine release, respectively.

CAR‐T cells have been shown to induce pyroptosis in tumor cells, a gasdermin‐mediated cell death pathway.^69^ In brief, CAR‐T cells trigger inflammation by secreting cytokines like TNFα and IFNγ, which upregulate gasdermin expression. Perforin released by CAR‐T cells induces pore formation in tumor cells, enabling granzyme A and B to enter, hydrolyzing gasdermin B or activating caspase 3 to cleave gasdermin E. The N‐terminal gasdermin fragment forms a pore in the plasma membrane, leading to leakage of damage‐associated molecular pattern (DAMPs), like ATP and heat shock proteins, and cytokines. Morphologically, pyroptosing cells have been described to swell with bubble‐like protrusions appearing on the surface of the cellular membrane before rupture, which is due to the influx of osmotic water.^70^ These DAMPs trigger pyroptosis in macrophages, a major contributor to CRS.^71^, ^72^ Therefore, targeting pyroptosis or DAMPs release could reduce inflammatory complications caused by CAR‐T cells.^51^, ^73^, ^74^ Our results indicate that reducing cytokine release, such as IFNγ, TNFα, as well as granzyme B, by SPIONs might be a promising therapeutic approach to control CRS.^51^ The reduced cytokine secretion prevented gasdermin expression and cleavage, thus, tumor cells are prevented from undergoing pyroptosis and instead undergo alternative cell death pathways, such as apoptosis (Figure 5). Importantly, the efficacy of tumor cell death induced by SPION‐loaded CAR‐T cells was maintained, except under conditions of low CAR density in combination with low CAR‐T cell numbers (Figure 3). As SPION‐loaded CAR‐T cells are expected to be magnetically enriched in the tumor region, tumor cell lysis will still be induced but shifted from inflammatory pyroptosis to apoptosis. This might reduce the overwhelming macrophage activation and mitigate systemic inflammatory side effects of current CAR‐T cells therapies.

Our study was conducted entirely in vitro, focusing on the effects of SPIONs on CAR‐T cells and their subsequent interaction with tumor cells in a controlled but limited environment. We targeted CSPG4, primarily expressed on melanoma cells, and performed short‐term experiments with transiently transduced CAR‐T cells. In clinics, CAR‐T cells are virally transduced for long‐term CAR expression over several generations. Whether our in vitro results will reduce inflammatory side effects such as CRS or neurotoxicity in clinical settings remains to be tested in vivo. A hematological tumor model treated with SPION‐loaded CAR‐T cells, without magnetic guidance, may provide initial insights of whether SPIONs can reduce the inflammatory side effects. For this purpose, a xenograft model such as immunodeficient NOD SCID gamma mice with human B‐cell leukemia will be treated with nonloaded or SPION‐loaded human CAR‐T cells. For magnetic targeting studies in solid tumors, larger allograft animal models need to be developed, as intra‐arterial delivery near the tumor site is required. Previously, mitoxantrone‐loaded SPIONs have been successfully enriched in the tumor of rabbits, resulting in partial or complete tumor remissions.^75^ For the targeted accumulation of cells, the same magnet will be applied, in combination with a robotic system, enabling the targeting of various positions within the tumor (Figure 6A). With this, we aim to specifically accumulate SPION‐loaded CAR‐T cells within the tumor region to improve CAR‐T cell therapy in solid tumors and minimize off‐tumor side effects. Additionally, MRI detection of SPION‐loaded T cells can enable noninvasive and nonradioactive monitoring of CAR.T cell trafficking and persistence in vivo (Figure 7).

## 4 MATERIALS AND METHODS

### 4.1 Isolation and cultivation of cells

Human T cells were isolated from blood of healthy volunteers or leukocyte reduction chambers (Transfusionsmedizin Erlangen) using the IBA CD3 Fab‐TACS® Isolation Kit (IBA, Germany), following the manufacturer`s instructions. T cells were cultured in RPMI 1640 medium supplemented with 10% heat‐inactivated (HI) fetal calf serum (FCS), 2% penicillin–streptomycin solution, 1% l‐glutamine, 1% amphotericin B (Gibco, USA), and 0.1 ng/mL IL‐7 (ImmunoTools, Germany). A375M cells and 293T cells were cultivated in RPMI 1640 medium supplemented with 10% HI‐FCS, 2% penicillin–streptomycin solution, 1% l‐glutamine, 1% amphotericin B. CRMM2 and UPMM3 cells were cultivated in RPMI 1640 medium supplemented with 10 HI‐FCS, 1%, l‐glutamine, 0.5% penicillin–streptomycin, 1 mM sodium pyruvate, 1× MEM nonessential amino acid solution, 1× MEM vitamin solution, 50 µM 2‐mercaptoethanol (Gibco). A253 cells were cultivated in McCoy's 5A medium (Gibco) supplemented with 10% HI‐FCS. Jurkat T cells were cultured in RPMI 1640 supplemented with 10% HI‐FCS, 2% penicillin–streptomycin solution, and 1% l‐glutamine. All cell lines were obtained from ATCC (USA) and cultured at 37°C in a humidified atmosphere with 5% CO_2_.

### 4.2 Endocytosis inhibition

CD3^+^ T cell were cultured at 1 × 10^6^ cells/mL. To inhibit individual endocytic pathways, cells were treated with 60 µM PitStop2, 80 µM Dynasore, 200 µM EIPA (Sigma–Aldrich, USA), or 40 µM Rottlerin (Merck Millipore, USA), for 30 min at 37°C, using 2% DMSO as a control. Then, T cells were loaded with 80 µg Fe/mL SPIONs for 4 h, washed twice with T cell medium to remove excess SPIONs, and cellular iron content was analyzed by AES. Iron content was normalized to untreated T cells prior to SPION‐loading from the same donor.

### 4.3 Visualization of SPIONs using STEM and EDX

STEM and EDX spectrum imaging was conducted at a Cs‐corrected Spectra (Thermofisher, USA) operating at 200 keV equipped with a SuperX G2 high‐sensitivity X‐ray spectrometer. The STEM micrographs were captured using a camera length of 98 mm and a HAADF detector. EDX spectrum images were recorded with a dwell time of 50–100 µs and a screen current of approximately 0.250 nA. Data processing and evaluation were performed using the Velox software (Thermofisher).

### 4.4 Flow cytometry

Flow cytometry analysis was performed using a Gallios flow cytometer and the Kaluza Analysis Software 2.1 (Beckmann Coulter, USA). Adherent cells were detached using Accutase (Innovative Cell Technologies, USA) before staining. Cells were washed twice with cold Ringer solution prior to staining. Extracellular antibody or viability staining was performed for 30 min at 4°C in the dark. T cell subsets were identified using anti‐CD4 and anti‐CD8 antibodies. Intracellular antibody staining was performed in permeabilization buffer (eBioscience, USA) for 40 min at 4°C after fixation with 4% formaldehyde for 15 min at room temperature. Before each step, at least two washing steps with cold Ringer solution was performed.

### 4.5 Electroporation of T cells with mRNA encoding a CSPG4‐specific CAR and loading with SPIONs

CSPG4‐specific CD3^+^ CAR‐T cells were generated by mRNA electroporation as described previously.^76^, ^77^ In brief, T cells, resuspended in Opti‐MEM (Gibco), were electroporated using a square‐wave pulse of 1250 V/cm for 5 ms at room temperature with 150 µg/mL CAR‐encoding mRNA. Nonelectroporated and Mock‐electroporated T cells, electroporated but without mRNA, served as controls. Electroporation efficiency was analyzed 4 and 24 h postelectroporation by staining T cells for CAR expression. After electroporation, T cells were seeded at 1 × 10^6^ cells/mL and loaded with 80 µg Fe/mL SPIONs in deionized H_2_O for 4 h, while control T cells received equivalent H_2_O volumes without SPIONs.

### 4.6 Impedance‐based analysis of tumor lysis

Relative CSPG4 expression was investigated by flow cytometry after staining with anti‐NG2‐PE (Invitrogen, USA), with dead cells excluded through AxV‐APC staining.

Tumor cell lysis was monitored over 96 h in 96‐well Agilent E‐plates using the xCELLigence Real‐Time Cell Analysis SP instrument (Agilent, USA). Optimal tumor cell concentrations/well were determined in prior experiments (not shown). A253 and CRMM2 cells we seeded at 7 × 10^3^ cells/well, A375M cells at 3 × 10^3^ cells/well, UPMM3 cells at 2 × 10^3^, and 293T cells at 5 × 10^3^ cells/well. After 24 h, SPION‐loaded CAR‐T cells were cocultured with the tumor cells at ratios of 20:1 or 5:1 in duplicates.

CAR expression of SPION‐loaded T cells was investigated 4 and 24 h posttransfection through flow cytometry using Goat F(ab’)2 anti‐human IgG‐RPE (Southern Biotech, USA). For comparable cytolytic efficacy of the SPION‐loaded CAR‐T cells, all tumor cells were seeded at 7.5 × 10^4^ cells/well. After 12 h, SPION‐loaded CAR‐T cells were cocultured with the tumor cells at ratios of 20:1, 10:1, or 5:1 in triplicates. Nonloaded CAR‐T cells and Mock‐electroporated T cells served as controls. Background impedance was measured through medium alone or with 80 µg Fe/mL SPIONs.

### 4.7 Analysis of cytokine secretion

SPION‐loaded CAR‐T cells were cocultured with 5 × 10^4^ A375M or 293T cells at 1:1 ratio for 16 h. Afterward, supernatant was collected and stored at −80° until analysis. Cytokine levels were measured using the bead‐based multiplex assay LEGENDplex (BioLegend, USA), according to the manufacturers protocol, and analyzed via flow cytometry. Data were evaluated using the LEGENDplex software Qognit (San Carlos, CA, USA).

### 4.8 Identification of pyroptosis via live cell imaging

SPION‐loaded or nonloaded CAR‐T cells were incubated with 5 × 10^3^ A375M cells at 5:1, 10:1, and 20:1 ratios. Mock‐electroporated T cells served as controls. Plasma membrane permeability was assessed by adding 1.1 µg/mL. After 10 h, cells were imaged using the Incucyte (Sartorius AG, Germany) live‐cell imaging microscope, and pyroptotic cells, identified by swelling, were counted per well.

### 4.9 Identification of Gasdermin D cleavage

For flow cytometry analysis of Gasdermin D cleavage, SPION‐loaded or nonloaded CAR‐T cells were incubated with 2 × 10^4^ A375M cells at a 10:1 ratio. Controls included Mock‐electroporated T cells and 293T cells. After 4 h, cells were detached and fixed overnight at 4°C in 4% formaldehyde, then permeabilized, stained with anti‐CD4, anti‐CD8, and anticleaved Gasdermin D (Cell Signaling Technology, USA), and analyzed by flow cytometry.

For western blotting, SPION‐loaded or nonloaded CAR‐T cells were incubated with 2 × 10^5^ A375M cells at a 10:1 ratio. Mock‐electroporated T cells were used as controls. After 4 h, cells were detached, lysed in 150 µL of RIPA lysis buffer containing 1× protease inhibitor cocktail (Roche, Switzerland), and kept on ice for 15 min. Afterward, lysates were homogenized by continuous passing through a 27G needle. Cellular debris was pelleted by centrifugation at 700 rcf at 4°C, the protein‐containing supernatant was transferred to fresh tubes and stored at −80°C. Protein concentration was determined by Bradford assay and 50 µg was loaded per lane onto a StainFree Gel (BioRad, USA). Prior to blotting, gels were activated for at least 2.5 min using Trans‐UV (302 nm) and then transferred onto a polyvinylidene difluoride (PVDF) membrane using a SemiDry‐Blotter (BioRad) for 15 min. Blocking and antibody incubation were performed in TBS‐T with 5% non‐fat dry milk. Membranes were incubated overnight at 4°C with GSDMD (1:1000; Cell Signaling; 39754S) or α‐Actinin (1:1000, Cell Signaling; 3134S) primary antibodies. HRP‐conjugated anti‐rabbit (1:500; Abcam; ab205718) secondary antibody was applied for 1 h at room temperature, followed by chemiluminescent detection using, Immobilon HRP substrate (Millipore) and a Chemidoc imaging system (BioRad).

### 4.10 Detection of SPION‐loaded T cells through 7 T‐MRI

Jurkat T cells were loaded over night with 80 µg Fe/mL, while controls received dH_2_O. At the next day, cellular iron content was measured as previously described. Cells were seeded in 0.2 mL PCR tube (Eppendorf, USA) at cell numbers corresponding to 5, 2.5, or 1.25 µg total iron in 125 µL. To this, 125 µL of 2% agarose solution was added, mixed thoroughly, and allowed to solidify in a prechilled PCR tube rack. The tubes were then embedded in a 1% agarose in 50 mL Falcon Centrifuge tubes (Fisher Scientific, USA). Imaging was conducted using a preclinical 7 T‐MRI (ClinScan 70/30; Bruker BioSpin, Ettlingen, Germany, gradient amplitude: 290 mT/m, slew rate: 1160 T/m/s) with a volume resonator radiofrequency coil (RF RES 300 1H 075/040 QSN TR; Bruker). Images were obtained using a 3D T_2_‐weighted turbo spin‐echo (echo time: 28.8 ms, repetition time: 5000 ms) and a 3D‐FLASH sequence (echo time: 18 ms, repetition time: 574.22 ms, flip angle: 40°), highlighting susceptibility differences in the samples.

High‐resolution 7 T‐MRI images were analyzed using the 3D slicer 5.6.2 software to measure scalar gray values within each tube.^78^ Scalar background values outside the PCR tube were subtracted from the sample scalar values.

### 4.11 Accumulation of SPION‐loaded CAR‐T cells in a dynamic flow system

To investigate the magnetic accumulation of SPION‐loaded CAR‐T cells under dynamic flow conditions, we used a peristaltic pump and μ‐Slide I Luer 3D channel slides (ibidi, Germany). CAR‐T cells were generated, loaded with SPIONs, and then stained with Hoechst 33342.

2 × 10^4^ A375M cells/well were seeded into the slide, incubated overnight and stained with Vybrant DiD (Thermofisher). Afterward, A375M cells were embedded in a fibrin gel matrix, consisting of 12 mg/mL fibrinogen with 100 U/mL thrombin (Sigma–Aldrich).

In a closed system, the slides were filled with media and 1 × 10^6^ CAR‐T cells were injected using a three‐way valve. A neodymium rod magnet (4 mm diameter, 25 mm height, adhesive force 670 g; Webcraft GmbH, Switzerland) was placed below the middle and outside chamber. The system ran at 9.6 mL/min for 1 h, followed by flushing with 10 mL of media. Slides were imaged using fluorescence microscopy.

### 4.12 Simulation of the magnetic field

The simulation of the magnetic field acting on the SPION‐loaded CAR‐T cells was performed as previously described.^79^, ^80^ In brief, COMSOL Multiphysics was used to simulate the magnetic field acting on the SPION‐loaded CAR‐T cells. The simulation solved the divergence of the magnetic flux density *B* and the dependence of *B* on the magnetic field strength *H*. As magnetic charge does not exist, the divergence of *B* is always zero.

∇·B=0.

The relationship between *B* and *H* is linear with the permeability *μ*

$$B=\mu H=\mu_{0}\;\mu_{r}H,$$

where $\mu_{0}=4\pi *10^{7}\frac{H}{M}$ and $\mu_{r}$ is the relative permeability of a medium relative to that of a vacuum. A homogeneous distribution of SPION loaded CAR‐T cells within the plate was assumed. The permeability (*μ* = 1.3) used was consistent with,^80^ although the well plate and magnet geometries and the magnet position are different, as previously described. Well plate geometries were based on the manufacturer's datasheet.

### 4.13 Spheroid infiltration by CAR‐T cells

Multicellular 3D‐spheroids were generated by coincubation of 1 × 10^4^ A375M cells with 1 × 10^3^ fibroblasts in an agarose‐coated 96‐well plate for five days. Spheroids cultured from 1 × 10^4^ 293T cells and 1 × 10^3^ fibroblasts, served as controls. SPION‐loaded CAR‐T cells were added at a 60:1 ratio and incubated with the spheroid with unloaded and Mock‐electroporated T cells serving as controls. After 36 h, microscopy images were taken. The spheroid area was analyzed in ImageJ, with a local threshold on the methods of Sauvola and Pietikainen to improve spheroid outlines detection.^81^

### 4.14 Statistical analysis

All data were processed in MS Excel (Microsoft, USA) with all statistical analyses being performed using Graphpad Prism 9.5.2 (San Diego, CA, USA). Data are shown as the mean ± standard deviation with at least three replicates, unless otherwise specified. Difference between groups were calculated using two‐way analysis of variance (ANOVA). Statistical *p* values ≤ 0.05 were considered as statistically significant.

## AUTHOR CONTRIBUTIONS

The manuscript was written through contributions of all authors. F. P., C. J., and C. A. conceptualized and planned the study. F. P. and C. J. wrote the manuscript. F. P., L. C., L. L., N. S., and J. D. performed experiments and F. P. analyzed the data. N. S. and J. D. produced the mRNA. M. L. and E. S. performed STEM and EDX. All authors have read and given approval to the final version of the manuscript.

## CONFLICT OF INTEREST STATEMENT

The authors declare no conflict of financial interests.

## ETHICS STATEMENT

All experiments were performed with approval from the institutional ethics review board of the Friedrich‐Alexander‐Universität Erlangen‐Nürnberg (reference number: 257_14B and reference number: 60_21B). The studies were conducted in accordance with the local legislation and institutional requirements. The participants provided their written informed consent to participate in this study.

## Supporting information



Supporting Information

Supporting Information

Supporting Information

## Data Availability

The datasets used during the current study are available from the corresponding author upon reasonable request. All data generated or analyzed during this study are included in this published article and its supplementary information files.
