# Supplementary material for: Loading of CAR‐T cells with magnetic nanoparticles for controlled targeting suppresses inflammatory cytokine release and switches tumor cell death mechanism
Source: MedComm (2020). 2025 Jan 5;6(1):e70039. doi: 10.1002/mco2.70039 (PMC11702464; doi:10.1002/mco2.70039)
Supplement: Supplementary file 1 — Supporting Information [file MCO2-6-e70039-s001.docx]

**Loading of CAR-T cells with magnetic nanoparticles for controlled targeting suppresses inflammatory cytokine release and switches tumor cell death mechanism**

Felix Pfister, Lucas R. Carnell, Lisa Löffler, Philipp Boosz, Niels Schaft, Jan Dörrie, René Stein, Malte Lenz, Erdmann Spiecker, Sami Haddadin, Carola Berking, Christoph Alexiou, and Christina Janko

**Physiochemical SPION characterization,** **Supplementary Figures 1-4, Supplementary Videos 1-2, legends, and references**

# Physiochemical SPION characterization

Investigation of the physiochemical features is needed to ensure reproducibility and comparability between batches. The mean magnetic susceptibility at an iron concentration of 1 mg Fe/mL was 4.10 ± 0.02, which was higher than in the original synthesis protocol by Elbialy *et al.* due to an increased iron(II) chloride to iron(III) chloride ratio to achieve a higher magnetic susceptibility and magnetizability.[1] The average hydrodynamic particle size [nm] in H_2_O was 54.3 ± 3.2, which is an optimal size for receptor mediated endocytosis,[2; 3] with a polydispersity index (PDI) of 0.149 ± 0.005. The zeta potential [mV] at a pH of 7.3 was -51.3 ± 2.6, indicating the negatively charged citrate shell. Absence of endotoxins was ensured as a prerequisite to use for loading of immune cells. The physicochemical features of three batches used in the actual experiments were very similar and in accordance with previous syntheses, proofing their reproducibility

# Materials and Methods

# Synthesis of SPIONs and physicochemical characterization

Three different charges of SPIONs were synthesized in-house based on an adjusted protocol of Elbialy.[1] Particles were sterilized by filtration through a 0.2 µm pore size filter. Absence of bacterial and endotoxin contaminations had been ensured by agar plate assays and an EndoZyme® II – Recombinant Factor C Endotoxin Detection Assay (BioVendor R&D, Brno, Czech Republic), which was performed according to manufacturer protocol. The reaction was monitored for 90 min at 37° C in 15 min intervals by recording the fluorescence at an excitation wavelength of 380 nm and an emission at 445 nm in a SpectraMax iD3 Plate reader (Molecular Devices, San José, USA). Spiking controls were carried out by adding a 5 µL of the highest standard to exclude assay interference.

Concerning their physicochemical features, SPIONs were analyzed regarding their size, iron content, magnetic susceptibility and zeta potential according to Mühlberger et al.[4] Their iron content in mg Fe/mL was investigated after diluting them 1:25 in deionized H2O, liquefying them in 65% nitric acid with atomic emission spectroscopy (AES), using Agilent 4200 MP-AES with an iron solution of 1000 mg Fe/L as an external standard (Bernd Kraft, Duisburg, Germany). SPION particles were diluted to the desired concentration with deionized water for all experiments.

# CAR-T cells proliferation and activation

CAR-T cells were co-cultured with either 5x104 A375M or 293T cells at a ratio of 1:1 in a 96-well plate. After 60 h, cells were investigated for proliferation, activation, cytokine production and after 120 h for differentiation. For proliferation, cells were fixed and permeabilized using the inside stain kit (Miltenyi Biotec, Germany) according to the manufactures protocol and stained with anti Ki67. Granzyme B expression was investigated similarly, however 1x Brefeldin A was added to the medium (eBioscience, USA) to maximize intracellular cytokine content. Activation of CAR-T cells was analyzed by extracellular staining with anti-CD25. T cell differentiation was investigated by staining with anti-CD45RO and anti-CD197.

# Analysis of viability and cellular iron content

Cell viability was assessed by flow cytometry at 4 h after SPION loading by staining with AxV-FITC, PI, and Hoechst 33342. Cellular iron content was quantified by AES using 2 x 106 T cells as described above.

# Label-free observation of tumor cell lysis

Tumor cell lysis was observed through label-free imager 3D Cell explorer fluo (Nanolive, Switzerland). SPION-loaded CAR-T cells were cocultured with 2x10^5^ A375M cells at a ratio of 20:1 in a 35 mm µ-dish (ibidi, Germany). The dish was kept inside a top-stage incubator ensuring stable humid conditions at 37° C and 5% CO_2_ during the whole imaging process. For 30 hours every 2 min a 3D picture with the Nanolive system was taken. Mock-electroporated, SPION-loaded T cells were monitored as control.

# Mycoplasma Detection

Mycoplasma contamination in cell culture was tested using the MycoAlert Mycoplasma Detection Kit (Lonza, Switzerland), according to the manufacturers protocol. Luminescence measurements were performed using the SpectraMax iD3 Plate reader.

# Cell line authentication

Cell lines were authenticated using the human cell line authentication service of Eurofins Genomics (Germany), according to the protocol of the manufacturer (not shown). For each cell line, at least 1 x 10^5^ cells were used.

# Supplementary Figures


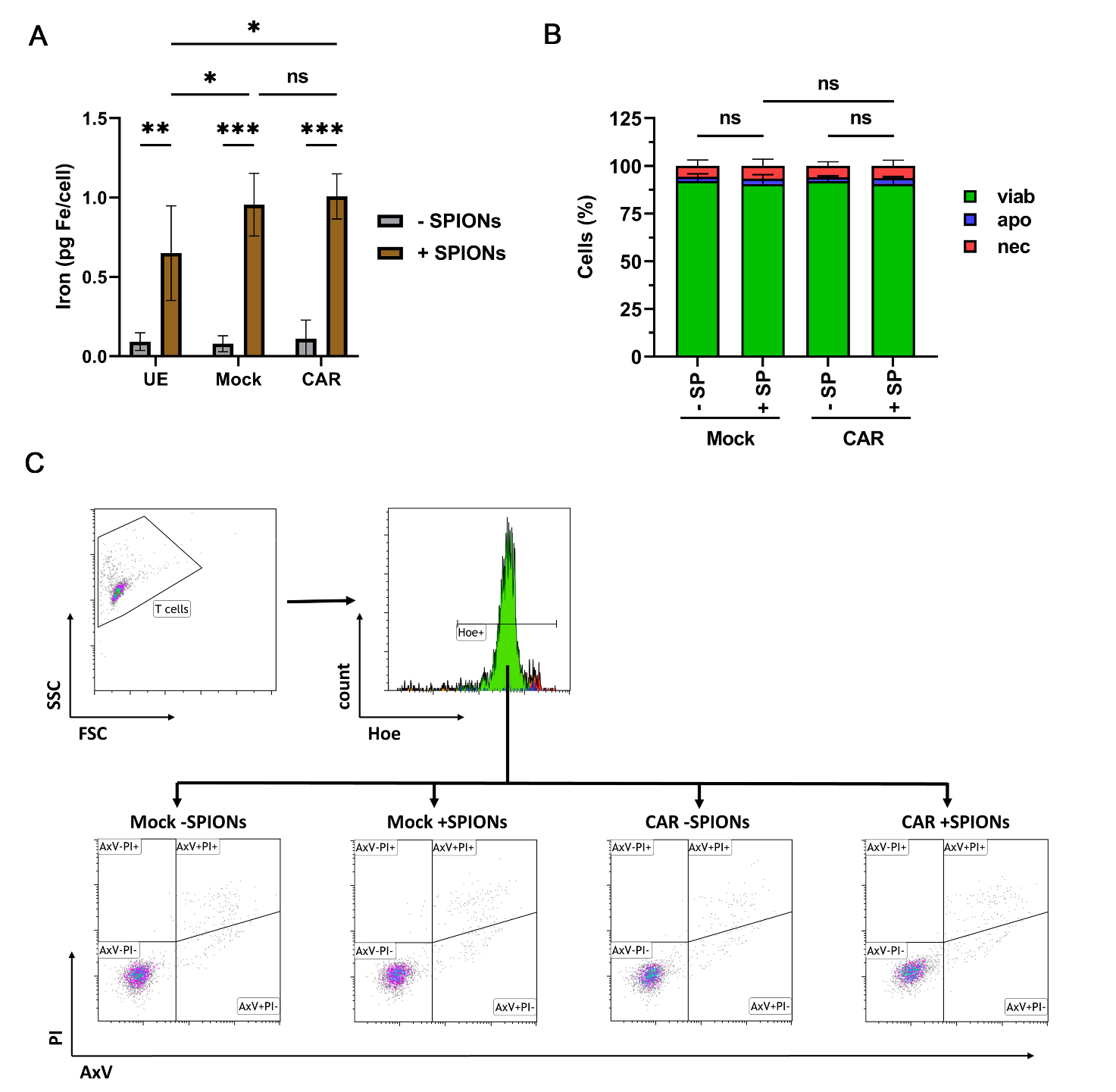


**Figure S1. Influence of electroporation on SPION uptake and T cell viability.** Isolated CD3+ T cells were not electroporated (UE), mock-electroporated without mRNA (Mock) or were transfected with mRNA encoding the CSPG4-specific CAR (CAR). T cells were loaded with 80 µg Fe/mL SPIONs (+ SPIONs) for 4 h, while dH2O was used as vehicle control (- SPIONs). A) Iron concentration per cell was determined using AES. B) The viability of T cells was determined by flow cytometry after staining with AxV and propidium iodide C) Representative flow cytometry plots of the gating strategy for analysis of T cell viability. T cells were identified via FSC and SSC, after which particle agglomeration were remsoved via Hoechst staining. Cellular viability was identified via AxV and PI, with AxV- and PI- cells were determined as viable, AxV+ and PI- as apoptotic, and AxV+ and PI+ as necrotic T cells. Significances (ns: non-significant; *p ≤ 0.05; **p < 0.01; ***p < 0.001; A) n = 3; B) n = 5) were calculated using a 2-way ANOVA. FSC: forward scatter, SSC: sideward scatter, Hoe: Hoechst.


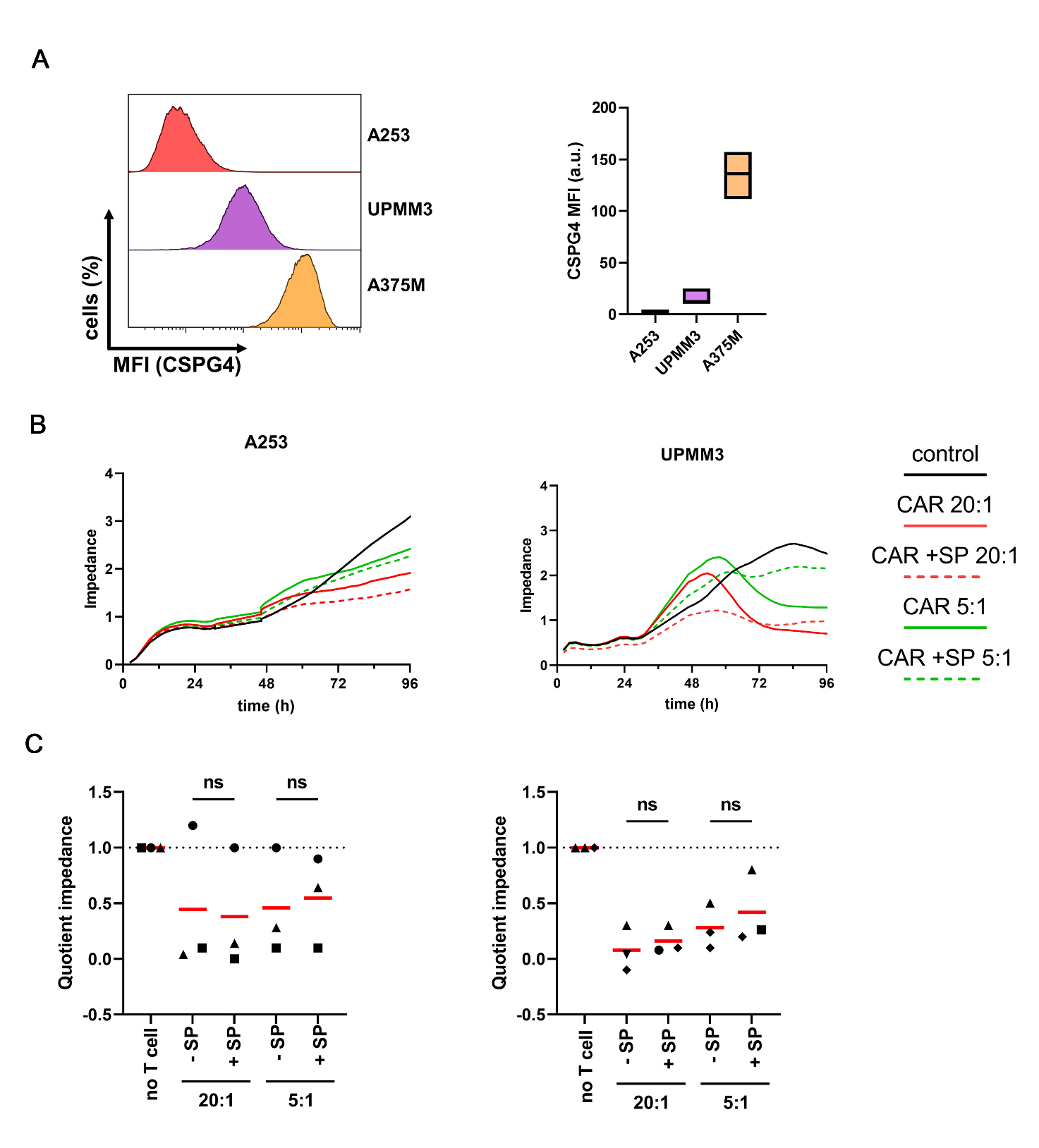


**Figure S2. CSPG4-specific melanoma cell lysis by SPION-loaded CAR-T cells.** Isolated primary CD3+ T cells received mRNA encoding a CSPG4-specific CAR. Afterwards, T cells were loaded with 80 µg Fe/mL SPIONS (+SP) for 4 h or dH2O as a control (-SP). Subsequently the T cells were co-incubated with the depicted 5*104 melanoma cells at ratios of 20:1 or 5:1. A) Exemplary MFI histogram of CSPG4 expression of the tumor cells and mean fluorescence intensity of CSPG4 on different tumor cell lines. Surface expression was determined by staining for CSPG4 and analyzed by flow cytometry. B,C) Killing of A253 or UPMM3 cells were monitored via xCelligence real-time impedance measurement. C) The quotient was calculated by division of the cell index of tumor cells without T cells after 96 h or at their respective maximum growth with the cell index of the corresponding condition. Experiments were performed in 3 independent experiments in duplicates with T cells from 3 donors, with 4 experiments from 4 donors for A375M. Impedance data from one exemplary donor each is shown. Significances (ns: non-significant; n = 3) were calculated using a 2-way ANOVA.


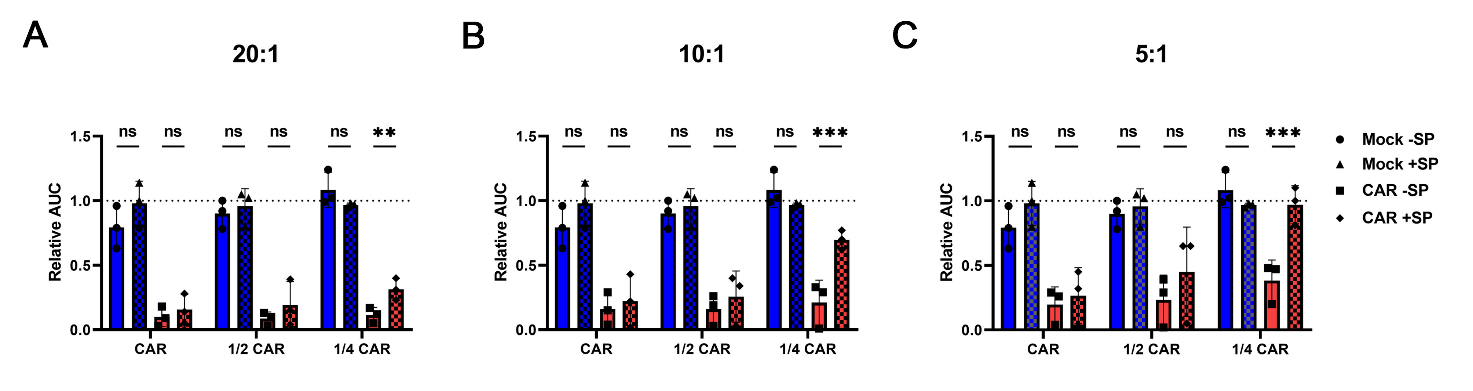


**Figure S3. Comparison of tumor cell lysis through Mock and CAR-T cells.** Isolated CD3+ T cells were electroporated without mRNA (Mock) or mRNA encoding a CSPG4-specific CAR, either with 150 µg/mL (CAR), 75 µg/mL (1/2 CAR) or 37.5 µg/mL (1/4 CAR). T cells were incubated with 80 µg Fe/mL SPIONs (+SP) for 4 h, or dH2O as vehicle control (-SP). Then, Mock or CAR-T cells were added to A375M cells in different T cell to tumor cell ratios (20:1, 10:1, 5:1). Killing of A375M cells was monitored via xCelligence real-time impedance measurement for 96 h. The AUC was normalized to the values of A375M cells without added T cells. Experiments were performed with T cells isolated from 3 donors. Significances (ns: non-significant; *p ≤ 0.05; ***p < 0.0001; n = 3) were calculated using a 2-way ANOVA. SP: SPIONs.


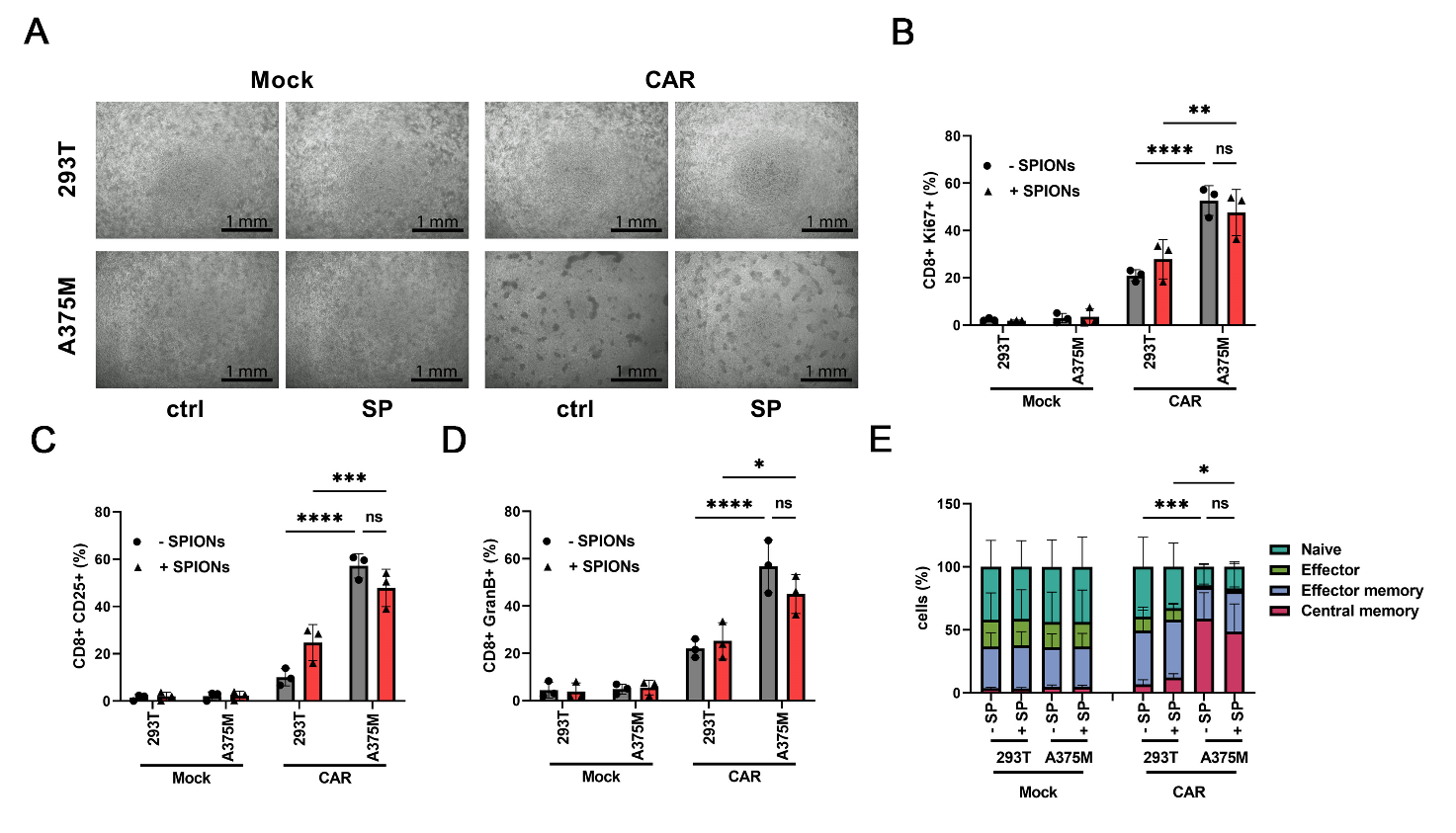


**Figure S4. Proliferation and activation of CSPG4-specific CAR-T cells after SPION-loading.** CD3+ T cells were electroporated with no mRNA (Mock) or mRNA encoding a CSPG4-specific CAR (CAR). Afterwards, T cells were loaded with 80 µg Fe/mL SPIONs for 4 hours and then incubated with 5*10^4^ A375M target cells or 293T control cells at ratio of 1:1. A) Proliferation clusters after 132 hours of incubation of T cells with tumor cells analyzed in microscopy. B) Ki67 expression was measured after 60 h. C,D) After 24 h, expression on the surface of CD8+ CAR-T cells of C) CD25 or D) intracellular Granzyme B was measured by flow cytometry. E) Differentiation of CD8+ CAR-T cells from naive T cells (CD45RO-/CD197+) to central memory cells (CD45RO+/CD197+), effector memory cells (CD45RO+/CD197-) and effector cells (CD45RO-/CD197-). Experiments were performed with T cells isolated of 3 independent donors. Significances (ns: non-significant; *p ≤ 0.05; **p < 0.01; ***p < 0.001; ****p < 0.0001; n = 3) were calculated using a 2-way ANOVA. ctrl: control; SP: SPIONs; GranB: Granzyme B.


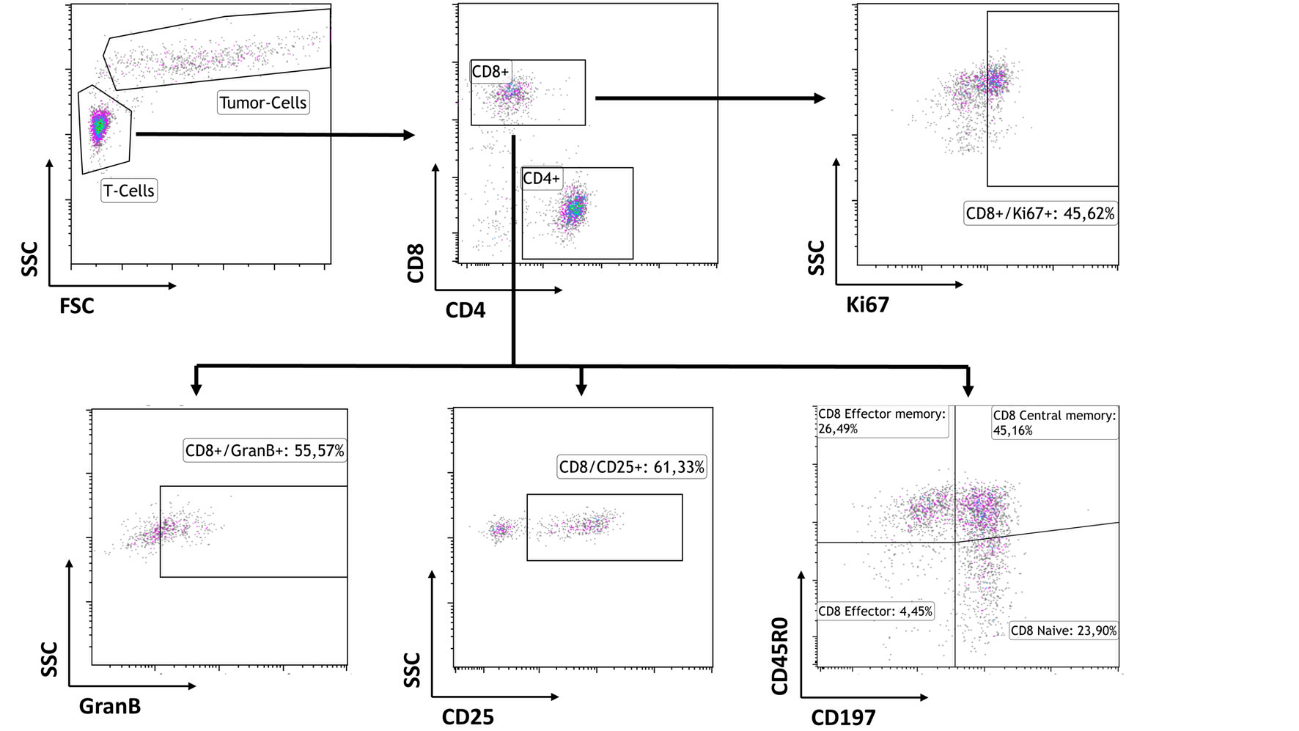


**Figure S5.** **Representative flow cytometry images of gating for T cell activation and differentiation.** Isolated CD3+ T cells were mock-electroporated without mRNA (Mock) or were transfected with mRNA encoding the CSPG4-specific CAR (CAR). T cells were loaded with 80 µg Fe/mL SPIONs (+ SPIONs) for 4 h (A,B) or overnight (C-F). dH_2_O was used as vehicle control (- SPIONs). T cells were identified via FSC and SSC, after which T cell subsets were identified by CD4 and CD8 staining. Proliferation of CD8+ T cells weas the identified via Ki67 staining, intracellular cytokine expression via Granzyme B staining, activation via CD25 staining, and differentiation via CD197 and CD45R0 staining. FSC: forward scatter, SSC: sideward scatter, GranB: Granzyme B.


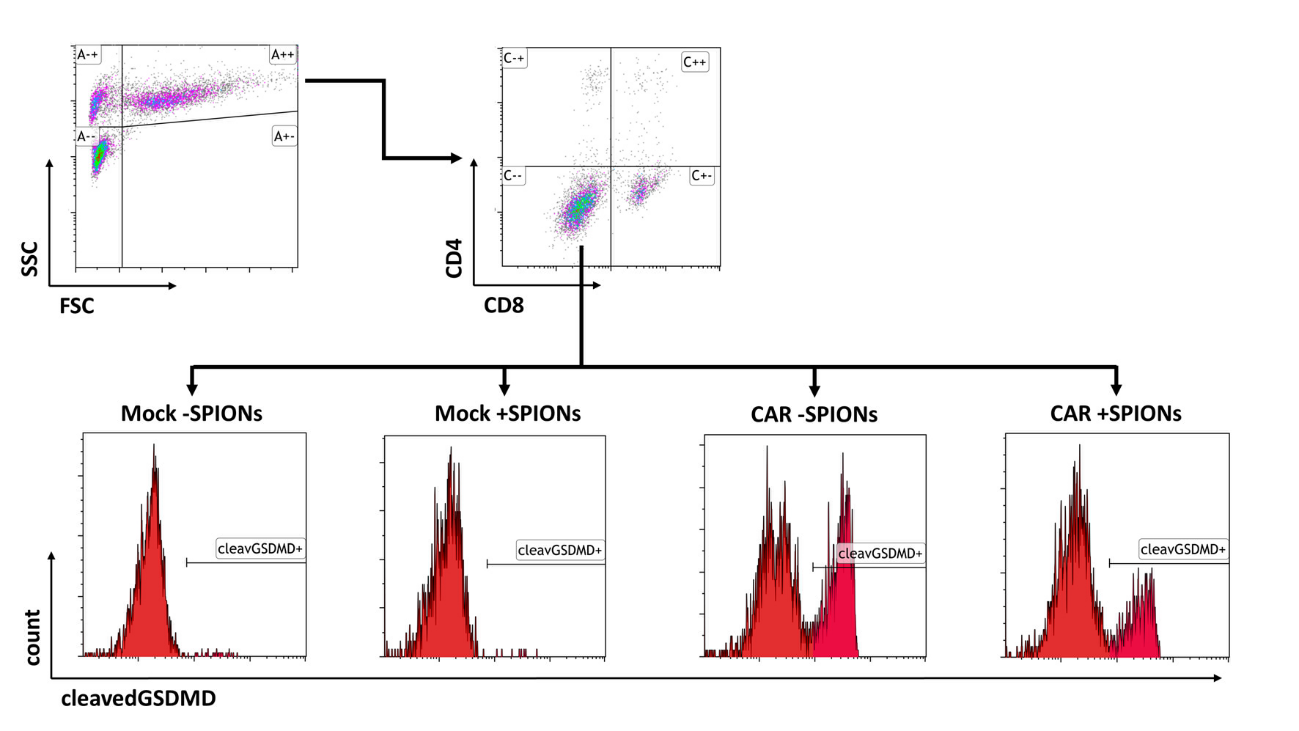


**Figure S6. Representative flow cytometry images of gating for Gasdermin cleavage.** CD3+ T cells were electroporated with no mRNA (Mock) or mRNA encoding a CSPG4-specific The T cells were then loaded with 80 µg Fe/mL for 4 h or with dH_2_O as a control. CAR. T cells were then co-cultured with A375M or 293T cells for4 h. The cells were then detached and fixed overnight in 4% formaldehyde. Single Tumor cells were identified via FSC and SSC, after which T cell subsets were excluded by CD4 and CD8 staining. Pyroptosis was identified via intracellular cleaved Gasdermin staining. FSC: forward scatter, SSC: sideward scatter, GSDMD: Gasdermin D.


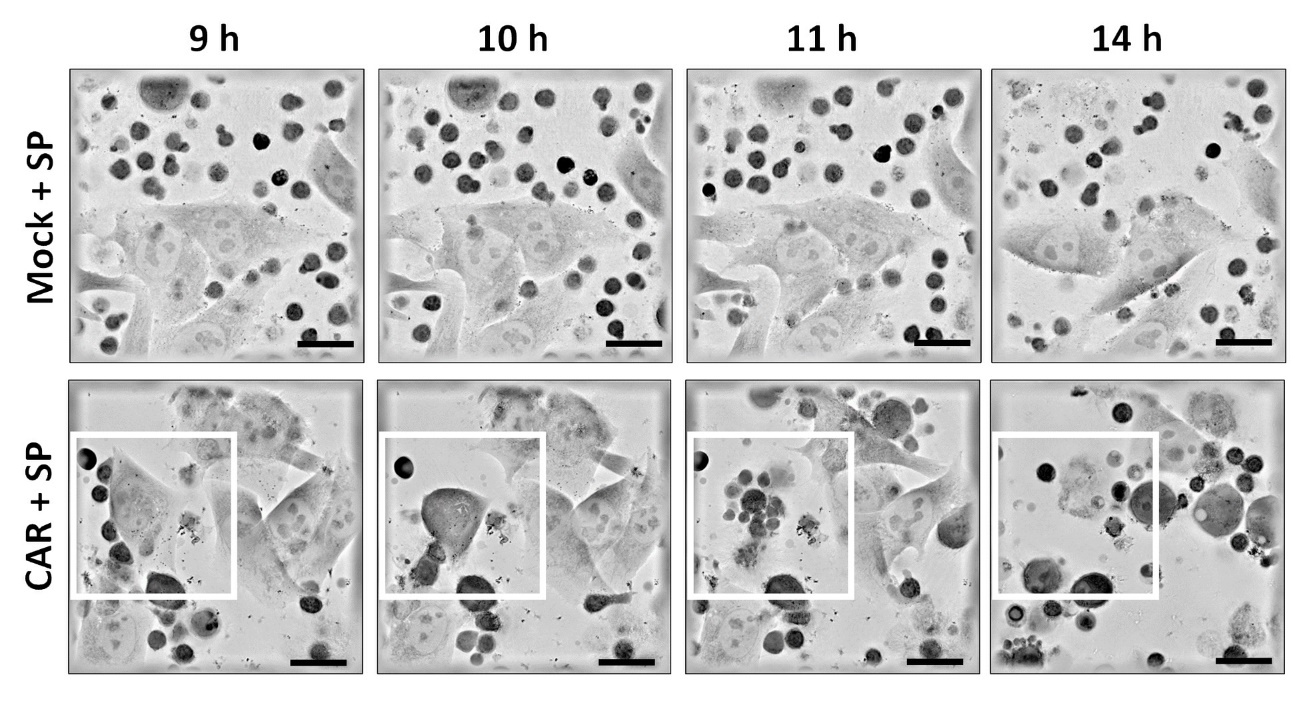


**Figure S7. Live cell imaging of tumor cell death.** Isolated CD3+ T cells were electroporated without mRNA (Mock) or with mRNA encoding the CSPG4-specific CAR (CAR). T cells were loaded with 80 µg Fe/mL SPIONs (+SPIONs) for 4 h, cells co-incubated with dH2O served as vehicle control (-SPIONs). Afterwards, the cells were incubated with 2+10^5^ A375M cells in a ratio of 10:1. Depicted are extracted images from Nanolive real-time microscopy. The black bar indicates 20 µm. SP: SPIONs


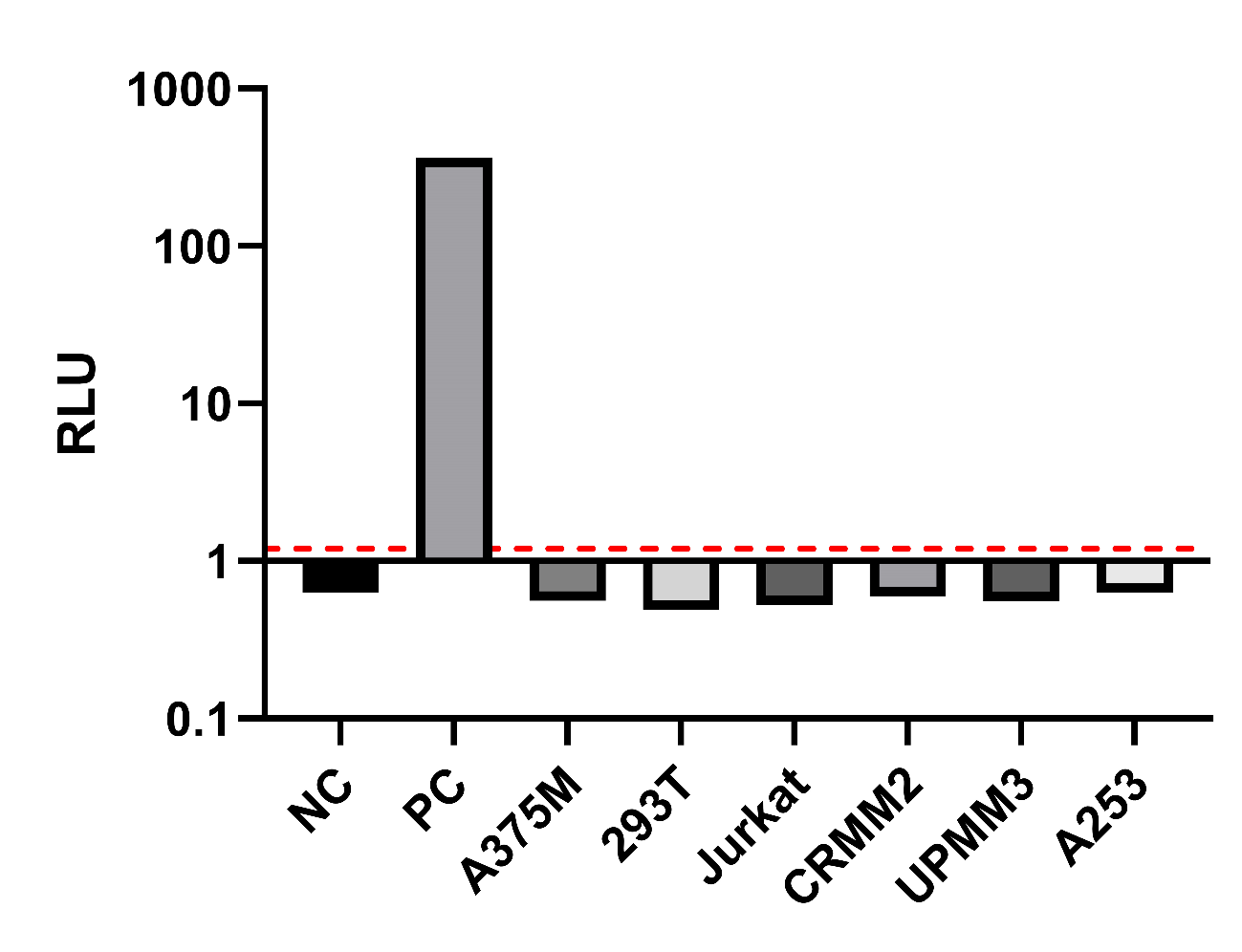


**Figure S8. Mycoplasma Detection.** Mycoplasma-free cell cultures were ensured through detection of mycoplasma-derived enzymes in the cell culture supernatant, with RLU values below 1.2 (dotted red line) indicating the absence of mycoplasma. N =1; NC: negative control; PC: positive control; RLU: Relative Light Units.

# Supplementary Videos

**Video S1. Live cell imaging of Mock-electroporated T cells.** Isolated CD3+ T cells were electroporated without mRNA (Mock). The T cells were then loaded with 80 µg Fe/mL SPIONs for 4 h, afterwards the T cells were incubated with 2*105 A375M cells at a ratio of 20:1. The cells were then imaged via the Nanolive real-time microscopy every two minutes. Shown is the 3D rendering of the cells over time.

**Video S2. Live cell imaging of CAR-electroporated T cells.** Isolated CD3+ T cells were electroporated with mRNA encoding a CSPG4-specific CAR. The T cells were then loaded with 80 µg Fe/mL SPIONs for 4 h, afterwards the T cells were incubated with 2*105 A375M cells at a ratio of 20:1. The cells were then imaged via the Nanolive real-time microscopy every two minutes. Shown is the 3D rendering of the cells over time.

# **References**

1 Elbialy, N. S., M. M. Fathy, and W. M. Khalil. 2015. "Doxorubicin loaded magnetic gold nanoparticles for in vivo targeted drug delivery." *Int J Pharm* 490 (1-2): 190-9.

2 McMahon, H. T., and E. Boucrot. 2011. "Molecular mechanism and physiological functions of clathrin-mediated endocytosis." *Nat Rev Mol Cell Biol* 12 (8): 517-33.

3 Pombo Garcia, K., K. Zarschler, L. Barbaro, J. A. Barreto, W. O'Malley, L. Spiccia, H. Stephan, and B. Graham. 2014. "Zwitterionic-coated "stealth" nanoparticles for biomedical applications: recent advances in countering biomolecular corona formation and uptake by the mononuclear phagocyte system." *Small* 10 (13): 2516-29.

4 Mühlberger, Marina, Christina Janko, Harald Unterweger, Eveline Schreiber, Julia Band, Christian Lehmann, Diana Dudziak, Geoffrey Lee, Christoph Alexiou, and Rainer Tietze. 2019. "Functionalization of T lymphocytes for magnetically controlled immune therapy: Selection of suitable superparamagnetic iron oxide nanoparticles." *Journal of Magnetism and Magnetic Materials* 473: 61-67.
